# scRNA-seq Reveals the Mechanism of *Fatty Acid Desaturase 2* Mutation to Repress Leaf Growth in Peanut (*Arachis hypogaea* L.)

**DOI:** 10.3390/cells12182305

**Published:** 2023-09-19

**Authors:** Puxuan Du, Quanqing Deng, Wenyi Wang, Vanika Garg, Qing Lu, Lu Huang, Runfeng Wang, Haifen Li, Dongxin Huai, Xiaoping Chen, Rajeev K. Varshney, Yanbin Hong, Hao Liu

**Affiliations:** 1 Guangdong Provincial Key Laboratory of Crop Genetic Improvement, South China Peanut Sub-Center of National Center of Oilseed Crops Improvement, Crops Research Institute, Guangdong Academy of Agricultural Sciences (GDAAS), Guangzhou 510640, China; dupuxuan2021@163.com (P.D.); dqq648841569@163.com (Q.D.); luqing@gdaas.cn (Q.L.); huanglu@gdaas.cn (L.H.); wangrunfeng@gdaas.cn (R.W.); lihaifen@gdaas.cn (H.L.); chenxiaoping@gdaas.cn (X.C.); 2 College of Agriculture, South China Agriculture University, Guangzhou 510642, China; wywang@scau.edu.cn; 3 WA State Agricultural Biotechnology Centre, Centre for Crop and Food Innovation, Food Futures Institute, Murdoch University (MU), Murdoch, WA 6150, Australia; vanika.garg@murdoch.edu.au (V.G.); rajeev.varshney@murdoch.edu.au (R.K.V.); 4 Key Laboratory of Biology and Genetic Improvement of Oil Crops, Ministry of Agriculture and Rural Affairs, Oil Crops Research Institute of Chinese Academy of Agricultural Sciences, Wuhan 430062, China; dxhuai@caas.cn

**Keywords:** scRNA-seq, peanut leaf, *FAD2*, oleic acids, gene atlases

## Abstract

*Fatty Acid Desaturase 2* (*FAD2*) controls the conversion of oleic acids into linoleic acids. Mutations in *FAD2* not only increase the high-oleic content, but also repress the leaf growth. However, the mechanism by which *FAD2* regulates the growth pathway has not been elucidated in peanut leaves with single-cell resolution. In this study, we isolated *fad2* mutant leaf protoplast cells to perform single-cell RNA sequencing. Approximately 24,988 individual cells with 10,249 expressed genes were classified into five major cell types. A comparative analysis of 3495 differentially expressed genes (DEGs) in distinct cell types demonstrated that *fad2* inhibited the expression of the cytokinin synthesis gene *LOG* in vascular cells, thereby repressing leaf growth. Further, pseudo-time trajectory analysis indicated that *fad2* repressed leaf cell differentiation, and cell-cycle evidence displayed that *fad2* perturbed the normal cell cycle to induce the majority of cells to drop into the S phase. Additionally, important transcription factors were filtered from the DEG profiles that connected the network involved in high-oleic acid accumulation (*WRKY6*), activated the hormone pathway (*WRKY23*, *ERF109*), and potentially regulated leaf growth (*ERF6*, *MYB102*, *WRKY30*). Collectively, our study describes different gene atlases in high-oleic and normal peanut seedling leaves, providing novel biological insights to elucidate the molecular mechanism of the high-oleic peanut-associated agronomic trait at the single-cell level.

## 1. Introduction

Peanuts (*Arachis hypogaea* L.) are a commercial crop used for edible oil and high-quality protein resources. The fatty acid (FA) composition of peanut seeds plays a decisive role in their nutritional value, edible quality, storage, and processing performance [1]. Oleic acid (OA, C18:1) and linoleic acid (LA, C18:2) are the main components of FA, accounting for approximately 80% of peanut oil. In modern breeding practice, market orientation anticipates the development of higher oleic acid (C18:1) varieties due to the antioxidant ability and storage stability of oleic acid, leading to the broad utilization of the *fatty acid desaturase 2* (*FAD2*) mutant as a donor in peanut breeding [2]. *AhFAD2* converts OA into LA by catalyzing the carbon dehydrogenation reaction, and mutation (*fad2*) results in peanut seeds with OA content exceeding 70% of total oil. Recently, the consumption of a high-OA diet has been proven to be associated with multiple health benefits [3]. Therefore, high OA is one of the most important breeding objectives of peanuts at present.

Plant FA are essential components of seeds and membrane lipids. They provide energy for various metabolic processes and participate in the development and stress response as signaling molecule precursors [4]. Although *fad2* induction increases the OA composition, it has been found to negatively affect peanut growth by causing dwarfing and reducing pod and seed size [5]. Moreover, the negative phenotype is also observed in *Arabidopsis*, where the *fad2* mutants displayed slightly delayed seed germination under cold temperature [6], and *fad2* was involved in the endoplasmic reticulum (ER) stress-induced growth repression [7]. Additionally, *FAD* family members mediating polyunsaturated fatty acids (PUFAs) are necessary for low-temperature survival, salt tolerance, and endoplasmic reticulum (ER) stress tolerance, which indirectly regulates plant growth characteristics [8]. A few studies have reported that the member of FAD protein-induced PUFA content affects plant development through cross-talk with the phytohormones pathway [9]. However, the comprehensive mechanism of *fad2* mutations regulating peanut growth and development has not been broadly elucidated, and understanding this mechanism is crucial for the application of *FAD2* during high-OA breeding.

With the development of cell isolation and high-throughput sequencing technologies, research on single-cell RNA sequencing (scRNA-seq) in plants has gradually increased [10]. Compared to the limitations of the traditional bulk transcriptome, scRNA-seq facilitates the study of intercellular gene expression heterogeneity and promotes the discovery of new cell types [11,12]. Recently, scRNA-seq was employed to define the developmental trajectories of root cells in *A. thaliana* [13,14] and to decipher the transcriptome profile of rice seedlings [15]. Although scRNA-seq has been successfully applied in these model plants, its use in non-model plants, especially for demonstrating cell development heterogeneity, is still limited.

High-OA peanuts have gained popularity among processing enterprises and consumers due to their high chemical stability and diverse beneficial effects on health [16]. However, during the process of popularization, it was discovered that the growth and development of peanut *fad2* seedlings, particularly the leaves, were delayed compared with normal peanuts. Since peanut leaves are critical for generating photosynthesis energy to increase the pod yield, it is important to understand the underlying biological mechanisms responsible for this delay. To address this, we developed a robust leaf individual cell isolation method and performed scRNA-seq to explore the cellular and transcriptional heterogeneity in high-OA peanut seedling leaf blades. Our study revealed that peanut *fad2* mutation restricted the leaf growth and cell differentiation through the hormone pathway at the single-cell resolution, providing novel biological insights into the molecular basis of high-OA peanut-connected growth development.

## 2. Materials and Methods

### 2.1. Plant Material, Growth Conditions, and Phenotype Assays

In this study, the high-OA variety, Yueyou271 (OA content > 70% of total oil), and the normal peanut, Yueyou43 (OA content > 45% of total oil), were investigated. Yueyou271 (with the *fad2* allele) is a near-isogenic line generated from the progeny population of a Yueyou43 hybrid with an *fad2* donor, Kainong176 [2]. The first generation of the high-oleic-acid peanut line (F1) continued to backcross with Yueyou43 (recurrent parent) to BC_8_F_2_ generation, and the positive line was determined with a molecular marker assistant, *FAD2* sequencing analysis, and oleic acid content examination. The Yueyou271 contains a similar genetic background to Yueyou43. The peanut seeds were sterilized in 1% sodium hypochlorite (NaClO) for 15 min, washed three times with sterile water, and sown in sterile soil. The seeds were grown in a growth chamber with a 14-h light (28 °C)/10-h dark (25 °C) cycle. The seedling’s phenotypic traits for leaf length, width, and area were investigated on days 3, 5, and 7 after sowing. The phenotypic data were recorded for 5 seedlings at each time point.

### 2.2. Protoplast Isolation and scRNA-seq Library Construction

Single-cell suspensions of one-week-old peanut seedling leaves were prepared as described previously [17]. Briefly, the leaf blade of one-week-old (7 days) peanut seedlings was cut into 1–2 mm strips and transferred into 30 mL of cellulase and pectinase enzyme solution (3% cellulose R–10, 0.3% pectinase, 1.5% macerozyme, 0.25% Bovine Serum Albumin, 5 mM MES, and 8% mannitol) isolate protoplasts. Protoplasts were then filtered with a 40 μm nylon strainer. The protoplast activity was detected by trypan blue staining, and then protoplast concentration and viable protoplast ratio were measured using a Countess^®^ II Automated Cell Counter (Thermo Fisher, Catalog Number AMQAX1000, Genedenovo company sponsor, Guangzhou, China). Then, the single-cell suspension was adjusted to the ideal concentration (≥1000 cells μL^−1^) in preparation for loading onto the chromium controller of the 10× Genomics platform. Further, 10× Genomics 3′ scRNA-seq libraries were constructed according to the user manual of the Chromium Single Cell 3′ Reagent Kit v3. Approximately 2 × 10^4^ isolated single cells were packed into gel bead-in-emulsions (GEMs) oil droplets. Then, the collected protoplasts were lysed, and the RNA of the GEMs droplet was reverse-transcribed into cDNA, followed by enzyme digestion, and PCR amplification. The constructed scRNA-seq libraries were sequenced with the paired-end mode of the Illumina sequencing platform.

### 2.3. Data Analysis of scRNA-seq of Peanut

Cell Ranger (version 3.1.0) [18] was used to generate a single-cell gene expression matrix and perform data quality statistics. Sequencing reads were aligned to the tetraploid peanut genome (GCF_003086295 available at peanutbase.org accessed on 14 June 2023) using the embedded STAR (Spliced Transcripts Alignment to a Reference) software [19]. Cell Ranger was then used to filter and correct barcodes and unique molecular identifiers (UMIs); only uni-mapped reads that aligned to only one gene were used for UMI counting. The cell barcodes with a total UMI count > 10% of the total UMI count of the 99th percentile of the expected number of recovered cells were screened to generate a gene expression matrix.

The Seurat R package (version 4.0.0) [20] was used for further cell filtration, normalization, cell clustering, and marker gene identification. Seurat normalized the count matrices after filtering the low-quality cells based on multiple quality control metrics to obtain highly variable gene sets. Canonical correlation analysis was carried out to correct the batch effect before merging data. Z-score normalization was performed on the merged data, followed by principal component analysis (PCA) for dimensionality reduction. Cell clustering and visualization were realized through nonlinear dimensionality reduction algorithms uniform manifold approximation and projection (UMAP) and t-distributed stochastic neighbor embedding (t-SNE). Up-regulated genes were screened by a likelihood-ratio test when comparing a single cluster to all other cells, of which the top five genes with the highest log_2_FC value were selected as cluster-specific marker genes.

Differentially expressed genes (DEGs) in different cellular clusters were mapped to GO terms in the Gene Ontology database (geneontology.org). Then, the GO terms that were significantly enriched in DEGs compared to the background genome were defined by a hypergeometric test. KEGG pathway enrichment analysis was used to identify the significant biochemical metabolic pathways or signal transduction pathways that are associated with DEGs. The expression profile and distribution of marker genes and DEGs were represented using heatmap and bubble plots, respectively. The sequences of DEGs were searched against the reference TAIR database (*Arabidopsis* genome) to obtain homologs in *Arabidopsis* to perform protein–protein interaction analysis using the STRING database [21] and Cytoscape (version 3.9.1) [22].

### 2.4. Marker Genes for Specific Cell-Type Validation

To identify the cell type, we first performed orthologous gene alignments of the reported marker genes in *Arabidopsis*. The marker gene list was downloaded from previous reports and three plant single-cell marker gene databases, including the PsctH [23], Plant Cell Marker [24], and PlantscRNAdb [25]. Furthermore, using the *Arabidopsis* marker genes as the query sequences, the homologous genes of the peanut were searched in Peanutbase.org. The top score hits were selected and annotated as the corresponding *Arabidopsis* cell type. This method determined the candidate marker genes, *Wox*, for the primordium cell and *FAMA* for the guard cell.

Cell-specific tissue isolation was performed according to the previously described method. Leaf were cut off mid-veins from blades as the vascular cells. The up-and-down epidermis layers were removed using tweezers as a mixed epidermis population and the mesophyll cells were isolated from the removed epidermis part by using a tweezer [26]. All samples were frozen in liquid nitrogen for total mRNA extraction and reverse transcription. RNA was extracted from each cell group to construct libraries by following the SMART-seq protocol (SMART-Seq HT Kit, Takara, San Jose, CA, USA). The cDNA library served as a template for detecting the gene expression level by applying conventional quantitative PCR with the ABI step one plus system. The epidermal cell population was used as the reference sample and the *Ah18S* was used as the internal reference control.

### 2.5. Pseudo-Time Trajectory Analysis

Pseudo-time trajectories were constructed by Monocle (version 3.0) [27] based on the dynamic expression pattern of key genes. The cells were ordered on a tree-like structure according to the changes of pseudo-time to simulate the cell differentiation relationship in the development process. Key genes related to the development and differentiation process were identified by analyzing the DEGs associated with the developmental trajectory, cell differentiation state, and cell fate. In addition, partition-based graph abstraction (PAGA) was used to arrange the low-dimensional projection positions of cells based on the similarity and dynamic change characteristics of gene expression patterns, which reconciled clustering and pseudotemporal ordering algorithms and allowed to infer complex cell trajectories [28].

### 2.6. RNA Velocity Analysis

RNA velocity analysis was performed by quantifying the spliced and unspliced reads using the Python script velocyto.py on the Cell Ranger output. The calculation of RNA velocity values for each gene in each cell and the embedding RNA velocity vector to low-dimension space were performed with the R package velocyto.R v0.6 [29]. Velocity fields were projected onto the UMAP embedding obtained in Seurat.

### 2.7. Cell Cycle Analysis

To perform the cell cycle analysis, the AddModuleScore function from Seurat was used to compute each cell dropped into the genome duplication phase by recounting the expression levels of cell cycle marker proteins in cell cycle analysis [30].

### 2.8. Phytohormones Uptake and Detection Assay

Leaf samples were collected at each time point, immediately frozen in liquid nitrogen, and ground into powder (30 Hz, 1 min). Powder (50 mg) for each sample was weighed into a 2 mL plastic microtube and dissolved in 1 mL of modified Bieleski’s solvent (methanol/water/formic acid = 15:4:1, *v*/*v*/*v*). A volume of 10 μL of internal standard mixed solution (100 ng/mL) was added into the extract as internal standards (IS) for the quantitation. The mixture was vortexed for 10 min, followed by centrifugation for 5 min (12,000 r/min, and 4 °C). Then, the supernatant was transferred to clean plastic microtubes, evaporated to dryness, dissolved in 100 μL 80% methanol (*v*/*v*), and filtered through a 0.22 μm membrane filter. LC-MS/MS was used for the qualitative and quantitative determination of phytohormone profiles. Each assay was performed in three replicates.

### 2.9. Tissue Real-Time PCR Analysis

Briefly, 1 µg of total RNA was reversely transcribed into cDNA using a PrimeScript RT Reagent Kit (Takara, Dalian, China) according to the manufacturer’s instructions. The PCR reaction was conducted in a 20 µL reaction system using SYBR Premix ExTaq™ (TaKaRa, Dalian, China) on an ABI StepOne Plus system. The relative expressions of target genes were calculated by the 2^−ΔΔ^CT method [31]. *Ah18S* was selected as an internal control. Each measurement was made with three biological replicates and data histograms with means ± SE.

## 3. Results

### 3.1. FAD2 Mutant Negatively Regulated the Growth Phenotypes of Peanut Seedling Leaf

**Figure 1:**
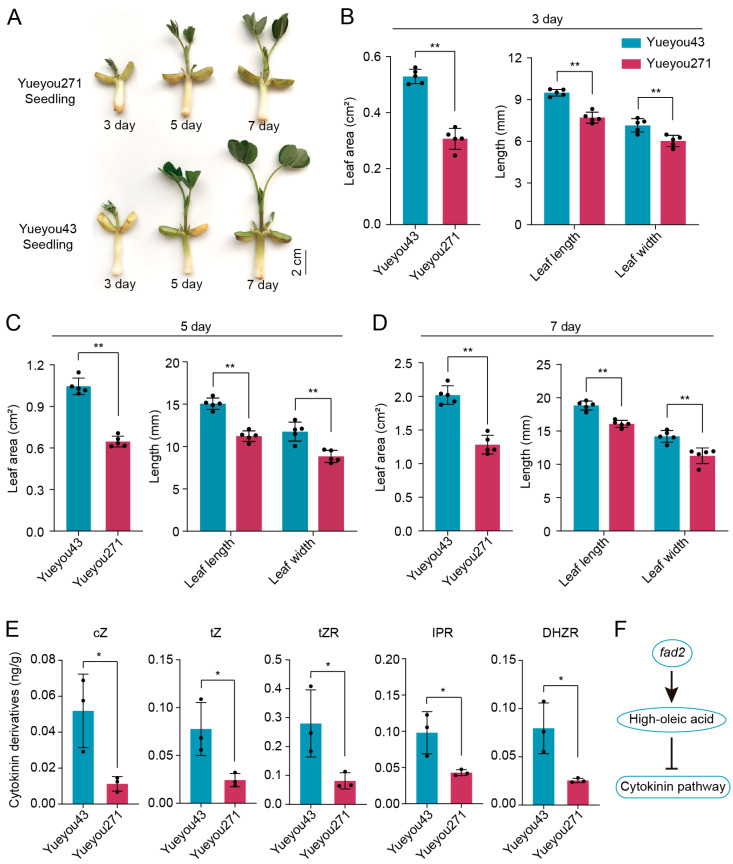
Phenotypic variations of peanut seedlings between high-OA cultivar Yueyou271 and normal-OA cultivar Yueyou43. (**A**) The growth phenotype of seedlings of the two varieties on day 3, 5, and 7 after sowing. (**B**–**D**) Leaf area, stem length, leaf length, and width of seedlings of the two varieties on day 3, day 5, and day 7 (*n* = 5). (**E**) Comparison of the cytokinin derivatives contents in seedling leaves between Yueyou43 and Yueyou271. Histograms depict the mean ± SD of three biological replicates. cZ, *cis*-Zeatin; tZ, *trans*-Zeatin; tZR, *trans*-Zeatin riboside; IPR, N6-isopentenyladenosine; DHZR, Dihydrozeatin ribonucleoside. The asterisks indicate significant differences between the two varieties (T-test, * *p* < 0.05, ** *p* < 0.01). (**F**) A model representing the high-OA accumulation mediated by *fad2* mutation to repress the cytokinin pathway.

The growth and development of high-OA variety Yueyou271 and normal peanut seedlings were compared under the same conditions and their phenotypic characteristics were documented on day 3, 5, and 7 after sowing (Figure 1A). A marked retardation in leaf growth was observed in the high-OA peanut Yueyou271, with its leaves tightly closed on day 5 and partially expanded on day 7. However, the leaves of normal variety (Yueyou43) were partially expanded on day 5 and almost completely expanded on day 7. Additionally, the leaf size of Yueyou271 was smaller than that of the normal peanut within one week. The results of the phenotypic investigation showed that the leaf length, width, and area of the high-OA variety were significantly lower than those of the normal peanut during the same period (Figure 1B–D). Therefore, the morphologic observations and phenotypic statistics of peanut seedlings demonstrated that the high-OA peanut repressed the leaf growth and development compared with the normal peanut.

Previous studies have demonstrated that the phytohormone pathway plays a critical role in regulating various processes of plant growth and development, including the expansion of leaf cells. In light of this, we collected seedling leaves from two peanut varieties to examine their phytohormone contents and observed that the levels of cytokinin, auxin, and GA contents were significantly lowered, whereas the levels of JA were significantly elevated in the high-OA peanut Yueyou271 compared to the normal peanut variety (Figure S2). In particular, *fad2* inducing the high-OA dramatically led to leaves failing to synthesize the *cis*- or *trans*-Zeatin (cZ/tZ) with a normal level, and the contents of cytokinin derivatives declined compared to the low-OA peanut (Figure 1E). Based on these findings, we hypothesized that *fad2* mutation may affect the phytohormone pathway, leading to the negative regulation of leaf tissue development in high-OA peanuts (Figure 1F).

### 3.2. scRNA-seq Identified the Major Cell Clusters in High-OA and Normal Peanut Seedling Leaf

**Figure 2:**
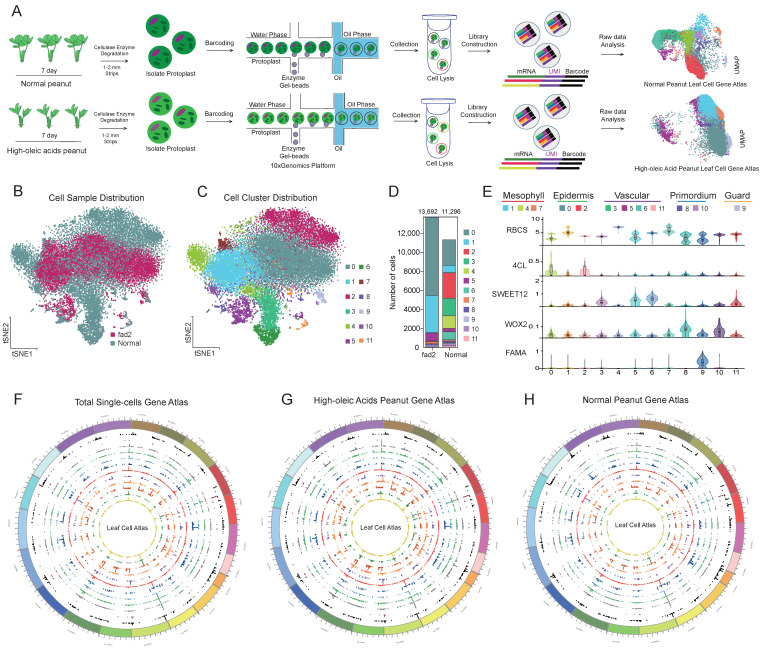
Construction of single-cell transcriptome atlas and annotation of peanut leaf clusters. (**A**) An overview of the scRNA-seq workflow used. (**B**,**C**) Visualization of 12 cell clusters using t-SNE plot; each dot indicates individual cells colored based on variety and cell clusters. (**D**) Bar plot depicting distribution of cells from high-OA (Yueyou271) and normal peanut (Yueyou43) in the 12 clusters. (**E**) Violin plots showing the expression pattern of known marker genes across clusters. (**F**–**H**) Circos plots consisted of 20 peanut chromosomes, representing the single-cell gene expression pattern in the leaf cells of total high-OA and normal peanuts, respectively. The outer circle to inner circle represents the cell clusters 0 to 11.

To investigate the association between transcriptional heterogeneity and delayed leaf growth in the *fad2* mutant, we performed single-cell transcriptome profiling using a microfluidic technology platform (Figure 2A). We obtained two gene expression matrices of 10,691 genes across 13,692 cells for the high-OA peanut and 10,395 genes across 11,296 cells for the normal peanut after aligning the raw sequencing data to the peanut genome and filtering low-quality cells (Tables S2–S4). Data quality control showed that a median of 1489 UMIs and 1074 genes and 2507 UMIs and 1461 genes were distributed in each cell of high-OA and normal peanuts (Figure S3A,B). Using *Arabidopsis* TFs as a reference, we identified 2568 and 2629 putative TFs for high-OA and normal peanuts, respectively (Tables S5–S7).

Following Z-score normalization and dimensionality reduction, ~25,000 cells from the two peanut varieties were classified into 12 distinct clusters using the Louvain method and subsequently visualized on t-SNE and UMAP plots (Figure 2B,C and Figure S5). The number of cells in each cluster ranged from 2 to 8225 for high-OA and from 85 to 2683 for normal peanuts (Figure 2D; Table S1). Interestingly, few clusters were enriched or specifically found in the two peanut varieties. The marker genes for each cluster were used to distinguish and annotate the different cell clusters (Figure 2E). The cluster-wise expression abundances of identified genes from the high-OA and normal peanut cells anchored onto the 20 peanut chromosomes shows cluster- and variety-specific gene expression (Figure 2F–H and Figure S4). The clusters 0 and 2 were classified as epidermal cells based on the enrichment of marker gene *4-COUMARATE COENZYME A LIGASE* (*4CL*), which plays a key role in the phenylpropanoid and lignin synthesis pathway [32]. Mesophyll cell clusters (clusters 1, 4, and 7) were marked by the expression of *RUBISCO BISPOSPHATE CARBOXYLASE SMALL SUBUNIT* (*RBCS*) involved in photosynthesis [33]. Vascular cells (clusters 3, 5, 6, and 11) were characterized based on the expression of *BIDIRECTIONAL SUGAR TRANSPORTER* (*SWEET*), which is specifically expressed in the phloem to mediate sucrose efflux [34]. The clusters 8 and 10 specifically expressed *WUSCHEL-RELATED HOMEOVOX* (*WOX*), which is a marker of primordium cells [35]. The guard cells marker gene *FAMA* (*FMA*) was exclusively expressed in cluster 9, which was clearly separated from other cell types on the t-SNE plot [36,37]. Taken together, all cell clusters were classified into five major cell types present in leaves, namely, mesophyll, epidermis, vascular, primordium, and guard cells, indicating that the peanut leaf is composed of highly heterogeneous cells.

### 3.3. Identification of Important DEGs in Distinct Leaf Cell-Types Revealed fad2 Mutation Repressed the Cytokinin Pathway

**Figure 3:**
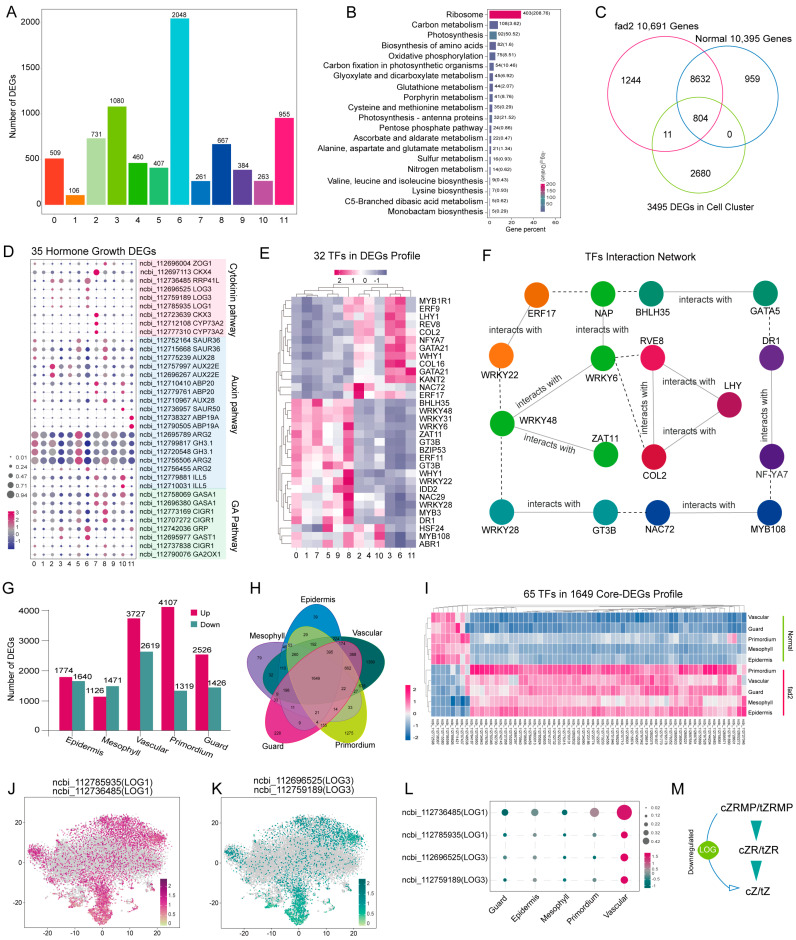
scRNA-seq identifies differentially expressed genes (DEGs) in distinct leaf cell types. (**A**) The number of DEGs identified in each cluster. (**B**) KEGG pathway enrichment analysis of all DEGs from all clusters. (**C**) Venn diagram showing the 804 core DEGs across all clusters. (**D**,**E**) Expression matrix of 35 hormone signaling DEGs and 32 differentially expressed TFs in each cell cluster. (**F**) The interaction network of 32 differentially expressed TFs. (**G**) Bar plots illustrating the up- and down-regulated DEGs in each cell type. (**H**) Venn diagram showing the 1649 core-DEGs across all cell types. (**I**) Heatmap depicting the expression level of 65 hub-TFs in each cell type of the two varieties. (**J**,**K**) The expression distribution of four *LOG* genes in all cell clusters, with the gray dots as background representing the cells with no expression of the given transcript. (**L**) Dot plots show the expression pattern and distribution of four *LOG* genes in distinct cell types. (**M**) A putative model illustrating that the down-regulation of *LOG* in Yueyou271 leads to a decrease in cytokinin content.

A total of 3495 significantly up-regulated DEGs were identified, which were distributed in the range of 106 to 2048 in distinct clusters (Figure 3A; Table S8). The top five genes with the highest expression level in each cell cluster were selected and their expression profiles were visualized in a dot plot (Figure S9; Table S9). Vascular cells (clusters 3, 5, 6, and 11) had the largest percentage of elevated DEGs. Furthermore, KEGG pathway enrichment analysis revealed that the cluster-specific DEGs were mostly involved in ribosome, carbon metabolism, and photosynthesis (Figure 3B). The 35 hormone DEGs related to cytokinin, auxin, and gibberellin metabolism were largely cluster-specific DEGs (Figure 3D). Meanwhile, 13 DEGs (Figure S10) were identified from the cell-cluster up-regulated DEGs profile (Table S8), which were involved in the JA biosynthesis pathway to reflect the up-regulated JA content in high-oleic peanuts. We further compared the expression profiles of cluster-specific DEGs and all identified genes of the two varieties and found a total of 804 core DEGs common to the two varieties (Figure 3C). Since transcription factors (TFs) recognize specific DNA sequences to guide the gene expression, we focused on the identification of critical TFs. We screened 32 TFs from the core DEGs and visualized their expression profiles using a heatmap (Figure 3E). Additionally, interactive network analysis of these 32 TFs showed that among these, 17 TFs consisted of protein–protein interaction networks (Figure 3F), including *LHY*, *COL2*, and *RVE8* responding to the photoperiod pathway; *ZAT11*, *WRKY* family gene, *NAC72*, and *BHLH35* being able to participate in the JA, abscisic acid (ABA), and salicylic acid (SA) activated stress defense reaction; and *ERF17*, *NF-YA7*, and *GATA5* being capable of regulating the growth hormone (IAA, CTK, GA) pathway. These TFs provide a gene resource to further validate their function in the development and differentiation state of the distinct leaf cell types. Furthermore, to determine the variation in gene expression in the two varieties, up-regulated DEGs and down-regulated DEGs from each cell type were examined by intergroup expression difference analysis (Figure 3G; Table S10). Then, 1649 core DEGs across cell types were assessed by comparing the above-mentioned DEGs, of which 65 TFs were subsequently selected and their expression profiles were described in a heatmap (Figure 3H,I). The transcript abundance of these 65 TFs displayed significant differences between the two varieties.

Furthermore, the core-DEG analysis revealed that four *LONELY GUY* (*LOG*) genes showed a higher abundance in various cell types of the normal variety (Yueyou43) compared to the high-OA peanut variety Yueyou271 (Figure 3J,K). LOGs exhibit phosphoribohydrolase activity, which directly converts the inactive cytokinin nucleotides, such as *cis*-zeatin riboside 5′-monophosphate (cZRMP) and *trans*-zeatin riboside 5′-monophosphate (tZRMP), into the active free-base form cZ and tZ [38,39]. The expression pattern of the four *LOG* genes in distinct cell types demonstrated that they were most highly expressed in the vascular cells (Figure 3L). To confirm these findings, we isolated the leaf veins and performed traditional real-time PCR at the tissue level, which showed that the expression levels of the four *LOG* genes were consistent with the scRNA-seq data (Figure S12A,B). As expected, the cytokinin (cZ/tZ) contents of the leaf vein were reduced in high-OA peanuts (Figure S12C). These results support the idea that the down-regulation of the *LOG* gene expression of high-OA peanuts causes a reduction of cytokinin content in vascular cells, ultimately leading to the growth and development retardation of its seedling leaves (Figure 3M).

### 3.4. FAD2 Mutation Repressed the Cell Differentiation in Leaf Development Trajectory

**Figure 4:**
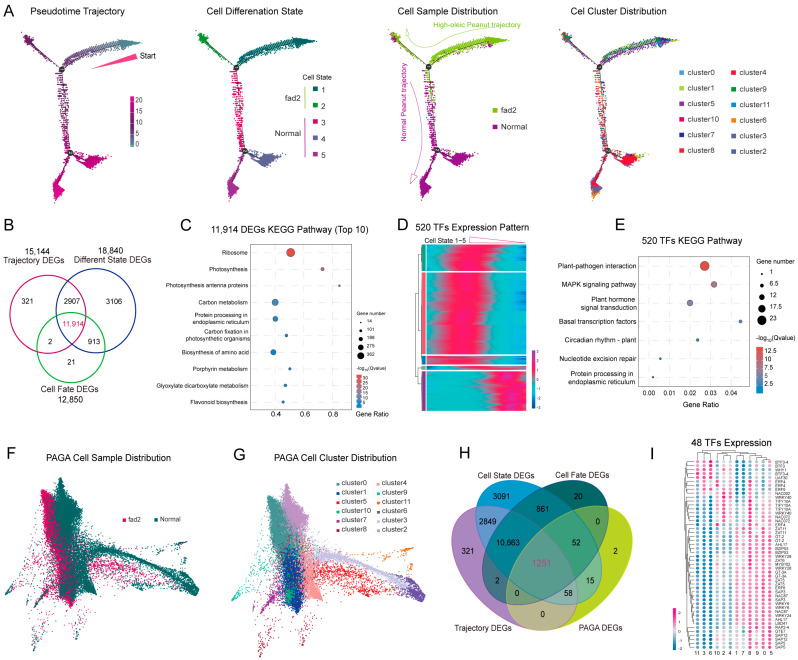
Pseudo-time trajectory analysis of cell types in one-week-old peanut seedling leaves. (**A**) The cell ordering along the differentiation trajectory presented by cell differentiation states, samples, and cell clusters. (**B**) Venn diagram showing the core DEGs from DEGs of cell differentiation trajectory, cell differentiation states, and cell fate. (**C**) KEGG pathway enrichment analysis of 11,914 core DEGs in leaf development trajectory. (**D**) Clustering and expression kinetics of 520 TFs in 11,914 core DEGs along cell differentiation states of total leaf cell ontology. (**E**) KEGG pathway enrichment analysis of 520 TFs in 11,914 core DEGs. (**F**,**G**) The cell ordering along the PAGA trajectory is presented by samples and cell clusters. (**H**) Venn diagram showing the 1251 core DEGs across both cell trajectories result. (**I**) Dot plots showing the expression pattern of 48 critical TFs in each cell cluster.

High-OA peanut exhibited slower leaf development than normal seedlings, implying that *fad2* probably affects the cell differentiation procedure. Therefore, pseudo-time trajectory analysis was employed to investigate the transcriptional difference between the high-OA and normal peanuts. This analysis showed that cells from high-OA peanuts collectively gathered in the cell differentiation states 1–2, whereas cells from normal peanuts were distributed in the total cell differentiation states 3–5 (Figure 4A). The cell sample distribution showed a differentiation dynamic deficient in high-OA cell trajectories compared to normal peanuts, suggesting that *fad2* mutation causing high-OA accumulation represses the cell differentiation and development in peanut leaves. Furthermore, 11,914 core DEGs were specifically involved in multiple biological pathways in the cell development trajectory (Figure 4B,C; Tables S11–S13). A total of 520 important TFs were filtered from the 11,914 core DEGs, which provided potential transcriptional dynamics for cell differentiation by participating in plant-pathogen, MAPK, and hormone signal pathways (Figure 4D,E).

To unbiasedly estimate the effectiveness of pseudo-time trajectory, PAGA (partition-based graph abstraction) was carried out to validate the correlation between distinct cell clusters in the development trajectory map. The *fad2* and normal peanut samples exhibited significant differences in PAGA trajectory (Figure 4F,G). Additionally, 1251 DEGs were screened by cross-comparing both cell trajectories, of which 48 critical TFs, consisting of the interaction network, probably dedicated the transcription dynamic to distinct cell type differentiation (Figure 4H,I and Figure S16; Tables S14 and S15). In conclusion, the identification of core DEGs provides a gene resource for illustrating the critical genes that modulate different processes between high-OA and normal peanut cell differentiation.

### 3.5. High-OA Peanut Regulated Cell Development Features in Distinct Cell Type Trajectories

**Figure 5:**
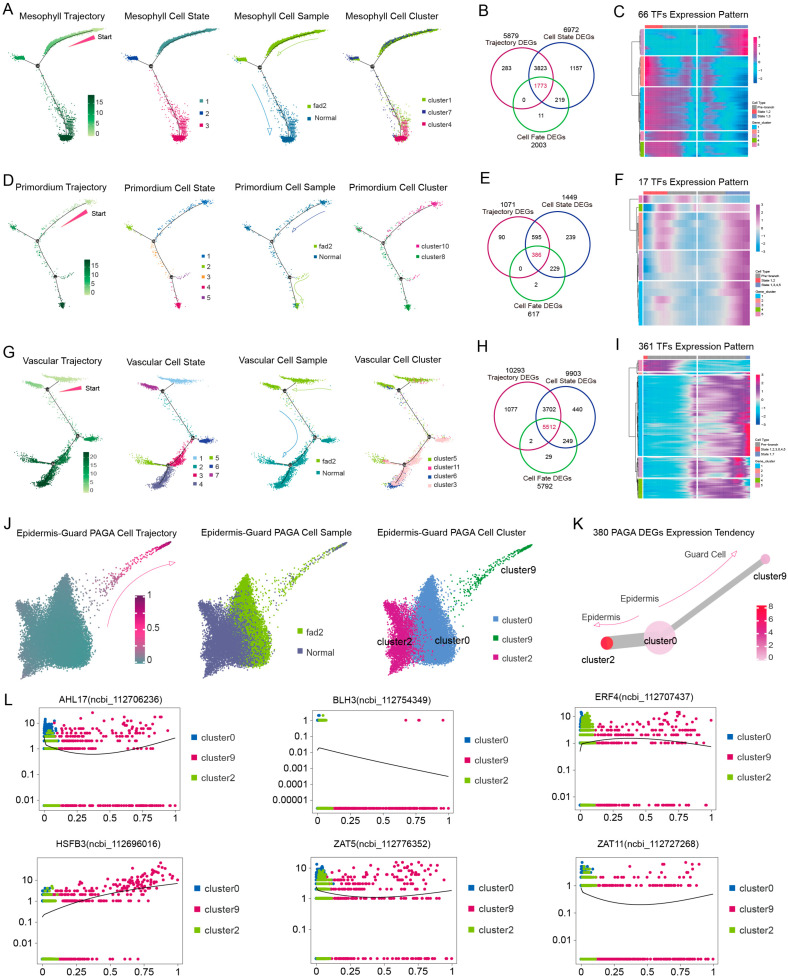
Separate development trajectory of five cell types. (**A**) Cell differentiation state, sample, and cell cluster distributions followed the pseudo-time trajectory of mesophyll development. (**B**,**C**) Clustering and expression kinetics of 66 TFs in 1773 DEGs along cell differentiation states of the mesophyll cell group. (**D**) Cell differentiation state, sample, and cell cluster distributions followed the pseudo-time trajectory of primordium development. (**E**,**F**) Clustering and expression kinetics of 17 TFs in 386 DEGs along cell differentiation states of the primordium cell group. (**G**) Cell differentiation state, sample, and cell cluster distributions followed the pseudo-time trajectory of vascular development. (**H**,**I**) Clustering and expression kinetics of 361 TFs in 5512 DEGs along cell differentiation states of the vascular cells. (**J**) PAGA trajectory of differentiation from the epidermal to the guard cells. (**K**) Expression tendency of 380 DEGs in PAGA trajectory. (**L**) Expression tendency of 6 TFs in 380 DEGs in the process of epidermis transforming into guard cells.

Mesophyll cells, the major group that carries out photosynthesis reaction due to their enrichment with chloroplasts, develop earlier in the high-OA seedlings than in normal seedlings. However, the *FAD2* mutation directly repressed the mesophyll differentiation by altering the cell distribution in the development trajectory map (Figure 5A). Meanwhile, 66 TFs were identified from the profile of 1773 DEGs profile, which showed expression trends that drove the differences in mesophyll differentiation at cell states 2–3 (Figure 5B,C; Tables S16–S18). In the primordium cell trajectory, the normal peanut cell originated earlier than the high-OA seedling; *fad2* mutation caused the primordium cell to lose the proliferative dynamic; and 17 TFs were featured, regulating the cell differentiation state 1–2 at branch node 1 (Figure 5D–F; Tables S19–S21). Moreover, we isolated the leaf vein that contained the vascular cells to examine the cytokinin content and this result suggested that cytokinin (*cis*-Zeatin, *trans*-Zeatin) content declined in the high-OA peanut (Figure S12C). The decrease in cytokinin content induced a repressed reaction of vascular differentiation in the high-OA cell, and the majority of TFs were down-regulated in the expression matrix of 361 different TFs profiles during the high-OA vascular cell differentiation to states 1 and 7, suggesting that the impact of the *fad2*-cytokinin module regulated cell ontology in vascular cells more so than other cell groups (Figure 5G–I; Tables S22–S24). Finally, epidermis-guard cells secrete the lipid molecules to cover the surface of the epidermis; due to the blocking of oleic acid converts to linoleic acid, the PAGA trajectory of epidermis-guard shows a noticeable difference of epidermis pavement cells developing into guard cells in high-OA peanuts (Figure 5J). Furthermore, six TFs were characterized from the 380 DEGs profile, which were involved in the ethylene and jasmonic acid (JA) pathways to regulate the process of the epidermis transforming into guard cells (Figure 5K,L; Table S25). These outcomes provide important biological insights into understanding how the *fad2* mutation causes high-OA repressed leaf growth at the level of subcellular types.

### scRNA-seq Reveals That High-OA Accumulation Represses Leaf Cell Cycle Procedure

**Figure 6:**
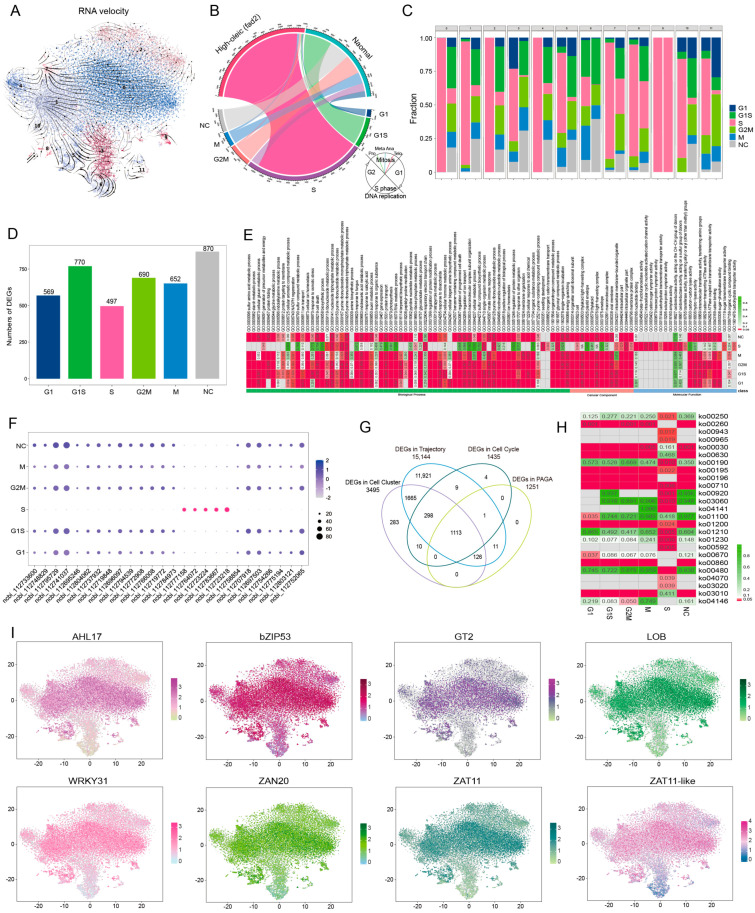
Cell cycle analysis provides important DEGs regulating cell cycle differences between the high-OA and normal seedling leaves. (**A**) RNA velocity analysis of all cells. The number 0–11 indicates the cell cluster 0–11 in the *fad2* and normal peanut seedling leaf t-SNE map. (**B**) Cell cycle phase distribution; NC indicates the non-cycling cell population. The scaleplate of out circular represents the total cell number in each cell cycle phase. (**C**) The histogram plot shows the distribution of cells from respective cell cycle phases. (**D**) The histogram plot shows the DEGs in different cell cycle phases. (**E**) GO enrichment analysis of DEGs in different cell cycle phases. (**F**) Newly identified genes for distinguishing leaf cell genome replication states. (**G**) Venn diagram showing 1113 core DEGs between cell-cycle-related DEGs, cell cluster DEGs, and cell development trajectory DEGs. (**H**) KEGG pathway enrichment analysis of 1113 core DEGs. (**I**) Cell expression distribution of eight core TFs identified from 1113 core DEGs.

The *FAD2* mutant significantly represses plant growth and development through regulation of the cytokinin pathway, which synergistically regulates the growth progression of cell cycle. Here, RNA velocity replicated that the gene transcription flow direction was weak in high-OA peanuts, whereas the transcriptional profile of the normal seedling cells showed a larger variation (diversity RNA flow arrows) (Figure 6A and Figure S17). Furthermore, cell cycle calling demonstrated that the number of cells which dropped into the cell dividing phase was larger than the non-cycling cells (NC) in the high-OA sample; in particular, the large proportion of cells dropping entirely into the S-phase in clusters 0 and 2 (epidermis), 4 (mesophyll), and 9 (guard cell) was greater than another cell cluster (Figure 6B,C). We next investigated the numbers of DEGs and the biological pathway they are involved in during phase G1 (569), G1S (770), S (497), G2M (690), M (652), and the non-cycling (870) phases of the cell cycle (Figure 6D,E; Table S26). A total of 26 DEGs were classified into a new marker group for future distinguishing leaf cell genome replication states, and 1113 DEGs were obtained to simultaneously modulate the cell cycle and cell development trajectory by involving in several pivotal metabolism pathways (Figure 6F–H). Finally, eight core TFs were identified in the 1113 DEGs profile, with significantly upregulated expression levels in high-OA peanut cells, potentially acting as downstream regulators to negatively mediate the cell cycle reaction (Figure 6I and Figure S18). This analysis provided a potential pool of TFs for further investigation of the functional details of critical genes regulating the cell cycle differences between high-OA and normal seedling leaves. Therefore, we hypothesized that the *fad2* mutant elevated the high OA content in the leaf blade, but excessive accumulation of oleic acid causes biological stress that suppresses the normal cell cycle procedure associated with plant growth.

## 4. Discussion

High-OA peanut oil is less prone to generating detrimental *trans*-fatty acids during storage and food processing due to its higher oxidative and thermodynamic stability, meeting the fast-increasing demand of consumers for nutritious and healthy edible oil [40]. Therefore, in addition to conventional breeding programs, some molecular breeding approaches such as RNA interference (RNAi), transcription activator-like effector nucleases (TALENs), and CRISPR/Cas9 systems have been utilized to reduce the activity of the *FAD2* genes and generate peanut lines with high-OA oil [5,41]. Although several efforts have been made in the development of high-OA peanuts, little is known about the impact of increased OA content in seeds on plant growth and development.

Recently, scRNA-seq has evolved as a technology, with a great potential to address complex biological questions with higher precision as compared to bulk RNA-seq [42]. High-throughput scRNA-seq can accurately dissect cell composition information and cellular heterogeneity with its high degree of resolution, providing new insights into plant physiology and development. In this study, we present the scRNA-seq atlas of the leaf blade of high-OA peanuts, which enabled us to explore its cellular and transcriptional heterogeneity and reveal the mechanism of the *FAD2*-regulated growth pathway at the single-cell resolution in peanut leaf development. Notably, the transcription factors interaction network identified by scRNA-seq provided a potential gene resource for understanding the difference in the metabolism pathway modulating cell development or differentiation determining growth differences between high-oleic acid and normal peanut seedlings. *WRKY6* [43] regulates the fatty acid composition and lipid accumulation; *ERF109* [44] and *WRKY23* [45] activate the growth hormone pathway; and *ERF6* [46], *MYB102* [47], and *WRKY30* [48] are potentially involved in leaf growth.

**Figure 7:**
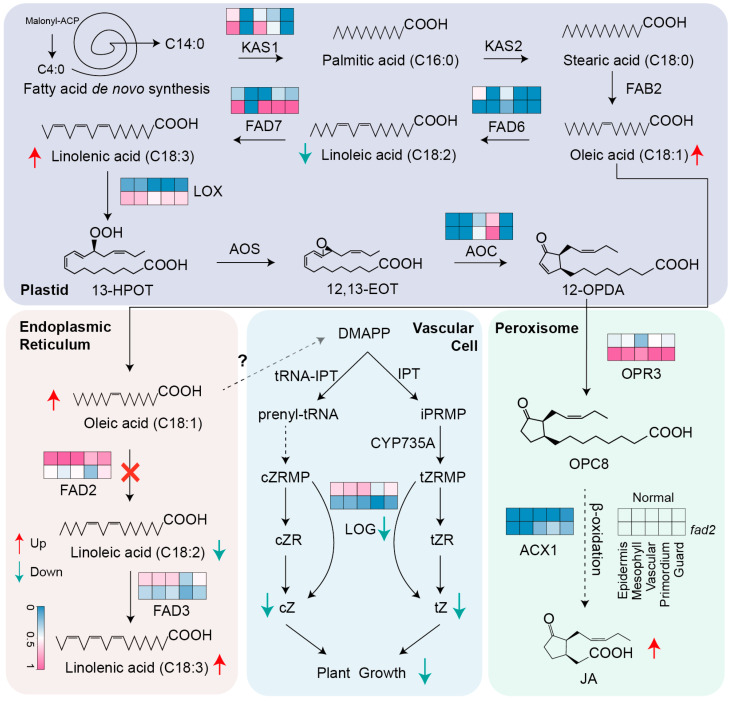
Putative model of *FAD2* mutant regulated the cytokinin pathway to repress the leaf cell development in peanut leaf vascular tissue. DMAPP, dimethylallyl diphosphate; iPRMP, N(6)-(Delta(2)-isopentenyl)adenosine-5′-monophosphate; cZRMP, *cis*-Zeatin riboside monophosphate; tZRMP, *trans*-Zeatin riboside monophosphate; cZR, *cis*-Zeatin riboside; tZR, *trans*-Zeatin riboside; cZ, *cis*-Zeatin; tZ, *trans*-Zeatin; 13-HPOT, 13-hydroperoxide of alpha-linolenic acid; 12,13-EOT, 12,13(S)-epoxy-9(Z),11,15(Z)-octadecatrienoic acid; 12-OPDA, 12-oxophytodienoic acid; OPC8, 3-oxo-2-(2-(Z)-pentenyl) cyclopentane-1-octanoic acid; JA, jasmonic acid; KAS1, 3-ketoacyl-acyl carrier protein synthase 1; KAS2, 3-ketoacyl-acyl carrier protein synthase 2; FAB2, stearoyl-ACP desaturase 2; AOS, allene oxide synthase; AOC, alleneoxide cyclase; ACX1, acyl-CoA oxidase1.

Fatty acid desaturase 2 (*FAD2*) is located in the endoplasmic reticulum and catalyzes the delta-12 desaturation reaction, which is a crucial step in the production of polyunsaturated fatty acids in oilseed crops. Understanding the regulation of the *FAD2* gene is important for comprehending fatty acid biosynthesis, plant development, and the essential role it plays in biotic or abiotic stresses [49]. *FAD2* not only regulates the conversion of oleic acid (C18:1) into linoleic acid (C18:2), but also its mutant increases the content of endogenous jasmonic acid (JA). However, evidences supports that the FAD protein induced PUFA content variation has an influence on plant development by cross-talking with the phytohormones pathway. Here, peanut *FAD2* modulated the antagonism relationship between the cytokinin (CTK) and jasmonic acid, as cytokinin down-regulation is mainly attributed to *fad2* deficiency in the endoplasmic reticulum, which restricts cytokinin biosynthesis. Furthermore, the peanut *fad2* mutant sightly up-regulates the content of linolenic acid (C18:3) by up-regulating the *FAD7* (*Fatty Acid Desaturase 7*) expression level that controls the plastid derived linoleic acid (C18:2) converts into linolenic acid (C18:3). The plastid yielded linolenic acid (C18:3) is a precursor in the JA synthesis pathway, thereby indirectly leading to an increase in the concentration of JA. Moreover, the expression levels of plastid *LOX* (*lipoxygenase*) and peroxisome *OPR3* (*12-oxophytodienoic acid reductase 3*) were up-regulated in the *fad2* mutant, indicating that high oleic acid probably improves the JA content by modulating the *FAD7*-*LOX*-*OPR3* tandem gene module (Figure 7). Next, the increased JA negatively works on cell proliferation and prevents the cytokinin response, and the inhibition of the JA biosynthesis enzyme will probably validate this hypothesis in our next study. Conclusively, the hormone disruption induced by the *FAD2* mutant reduces peanut growth characteristics, which may provide a potential reference for deciphering the yield decrease caused by small seeds in actual high-oleic peanut cultivation. In future breeding practice, artificial synthetized cytokinin enzymes’ coding sequencing can be inserted into the peanut genome with the genetic background of *fad2* mutation. Like the gene pyramiding of *CYP735A* (cytochrome P450) and *LOG1* improves the endogenous cytokinin content by transgenic-bio-technique-induced gene staking, this method may be able to rescue the growth hormone decrease in high-oleic peanuts. Moreover, the application of exogenous plant growth regulators with a suitable concentration of cytokinin probably benefits the high-oleic peanut yield, increasing in the field cultivation management.

High oleic acid (*fad2*) represses the peanut seedling leaf development by reducing the growth cytokinin and its derivatives contents; this reaction occurs in the vascular cell with a lower expression level of cytokinin synthesis, restricting the enzyme LOG through scRNA-seq identification, implying that *fad2* may produce a negative effect on peanut vascular system development to repress whole plant growth. Meanwhile, the cell development trajectory constructed using scRNA-seq data indicated that *fad2* suppressed cell differentiation, including the mesophyll and epidermis cell distributed limiting cell differentiation states, which seemed to lose the differentiation dynamic in pseudotime trajectory. Compared to conventional bulk RNA-seq, scRNA-seq analysis allows for the identification of cell type-specific DEGs. The gene network constructed using the single-cell data allows the identification of the critical biological pathway to elucidate the molecular mechanism of high-oleic-peanut-associated agronomic traits.

The cell cycle is a series of events that take place in a cell as it grows and divides. A cell spends most of its time in interphase, and during this time it grows, replicates its chromosomes, and prepares for cell division. The cell then leaves interphase, undergoes mitosis, and completes its division. The resulting cells enter their own interphase and begin a new round of the cell cycle. In this study, scRNA-seq data suggested that the majority of high-oleic peanut cells dropped into the S-phase during leaf growth. This result provides a novel insight for exploring the *FAD2* mutant’s control of high-oleic acid accumulation, which may influence downstream DNA replication events. Therefore, cell cycle disorders might play an intermediary role between high-oleic acids and eventual morphological changes in growth. Additionally, scRNA-seq analysis of gene expression patterns in high-/normal-oleic acid peanut seedlings has provided a gene resource for further illustrating the homologous DEG-transcription-mediated cell cycle difference in the *fad2* mutant, especially the transcription factors that respond to hormone pathways and modulate DNA-replication-related chromatin states.

Plant single-cell RNA-seq is a powerful platform that allows for the construction of cell atlases with a single-cell resolution [50,51]. However, the current method of isolating single cells from plant tissues depends on the cellulase-pectinase-based enzymatic degradation of the cell wall to obtain protoplasts and is associated with a microfluidic platform to construct the scRNA-seq library. The use of protoplasts as a biological material to describe the gene expression atlas is challenging due to the presence of cellulose and lignin in the cell walls that resist degradation [52]. To overcome the limitations of protoplast dissociation, an alternate method has been proposed that utilizes single nuclei to obtain a transcriptional profile, as transcription occurs in the nucleus and mRNA is exported into the cytoplasm for translation [53,54]. scRNA-seq can be replaced by single-nucleus RNA sequencing (snRNA-seq) to explore single-cell multi-omics research in future. Despite single-cell gene expression, atlas has been established in high-oleic peanut leaves, whereas high-oleic peanut pods and seeds are the most important tissues for harvest; therefore, illustrating the fatty acid or protein synthesis gene expression patterns at single-cell resolution should be listed in our schedule, and it is necessary to develop the snRNA-seq based on single-nuclei isolation and fluorescent-activated cell sorter (FACS) in peanuts. With the development of biotechnology, we anticipate incorporating various scRNA-seq/snRNA-seq-compatible methodologies, particularly in combination with single-cell spatial transcriptomics, which can overcome some of the limitations of traditional scRNA-seq by preserving in situ gene expression profiling. Future studies will involve deeper coverage in single-cell multiomics sequencing, which is based on the integration of individual-cell epigenetic landscapes for instant scATAC-seq, scChIP-seq, scCUT&Tag, scATAC-seq, and transcription atlases in the procedure of allopolyploid organ development. In the future, novel biotechnology at the single-cell level can accelerate the advancement of plant single-cell sequencing and provide insights into previously unexplored mechanisms of plant development.

## Data Availability

The raw sequencing data have been deposited to the China National Center for Bioinformation (CNCB) under bio-project accession CRA010421.

## Supplementary Materials

The following supporting information can be downloaded at: https://www.mdpi.com/article/10.3390/cells12182305/s1, Figure S1: Coding sequence of *FAD2-A* and *FAD2-B* in Yueyou271 (high-oleic-acid peanut) and Yueyou43 (normal-oleic-acid peanut); Figure S2: Phytohormone detection results in seedling leaves of Yueyou43 and Yueyou271; Figure S3: scRNA-seq raw data quality control; Figure S4: Circos plots depicting the single-cell gene expression pattern in leaf cells of total transcription factors (TFs); Figure S5: Visualization of 12 cell clusters using UMAP plot; Figure S6: The validation of scRNA-seq data of the five marker genes; Figure S7: Go enrichment analysis of all DEGs; Figure S8: KEGG pathway enrichment analysis of all DEGs; Figure S9: Dot plot depicting top five marker genes for each cell cluster and the cell expression distributions of two TFs (*COL16* and *NFXL1*) in top five genes profile; Figure S10: DEGs involved in the JA biosynthesis pathway; Figure S11: Interaction network of TFs constructed using Arabidopsis homologues of the 65 TFs in 1649 core DEGs profile; Figure S12: The validation of the scRNA-seq data in distinct cell populations; Figure S13: The relative expression of the top ten DEGs in the whole differentiation trajectory, cell differentiation branch 1 and branch 2; Figure S14: Expression tendency of 1300 DEGs in PAGA trajectory; Figure S15: The relative expression of top ten DEGs in PAGA trajectory; Figure S16: Interaction network of TFs constructed using *Arabidopsis* homologues of the 48 TFs in 1251 core DEGs profile; Figure S17: RNA velocity analysis of seedling leaf cells of high-OA peanut and normal peanut; Figure S18: The validation of scRNA-seq data of the eight critical TFs identified from 1113 core DEGs; Table S1: Cell number in each cell cluster; Table S2: All identified genes; Table S3: All genes in *fad2* peanut; Table S4: All genes in normal peanut; Table S5: All TF in both sample; Table S6: All TF in fad2 peanut; Table S7: All TF in normal peanut; Table S8: All DEGs in each cluster; Table S9: Top five DEGs in each cluster; Table S10: DEGs in both samples; Table S11: DEGs in trajectory; Table S12: DEGs in cell state; Table S13: DEGs in cell fate; Table S14: All PAGA DEGs; Table S15: 1251 PAGA DEGs; Table S16: DEGs in mesophyll trajectory; Table S17: DEGs in mesophyll cell state; Table S18: DEGs in mesophyll cell fate; Table S19: DEGs in primordium trajectory; Table S20: DEGs in primordium cell state; Table S21: DEGs in primordium cell fate; Table S22: DEGs in vascular trajectory; Table S23: DEGs in vascular cell state; Table S24: DEGs in vascular cell fate; Table S25: DEGs in PAGA trajectory of epidermis-guard; Table S26: DEGs in cell cycle.
