# Supplementary material for: scRNA-seq Reveals the Mechanism of Fatty Acid Desaturase 2 Mutation to Repress Leaf Growth in Peanut (Arachis hypogaea L.)
Source: Cells. 2023 Sep 19;12(18):2305. doi: 10.3390/cells12182305 (PMC10527976; doi:10.3390/cells12182305)
Supplement: Supplementary file 1 [file cells-12-02305-s001.zip › cells-2478484-Supplementary Figures.pdf]

Supplementary Files

>*FAD2-A* coding sequence in high-oleic cultivar Yueyou271
ATGGGAGCTGGAGGGCGTGTCACTAAGATTGAAGCTCAAAAGAAGCCTCT
TTCAAGGGTTCCACATTCAAACCCTCCATTCAGTGTTGGCCAACTCAAGAA
AGCAATTCCACCACATTGCTTTGAACGTTCTCTTTTCATATCATTCTCCTATG
TTGTCTATGATCTCTTAGTGGCCTACTTACTCTTCTACATTGCCACCACTTAT
TTCCACAAGCTTCCATACCCATTTTCCTTCCTTGCTTGGCCAATCTATTGGGC
CATCCAAGGCTGCATTCTCACTGGTGTTTGGGTGATTGCTCATGAGTGTGG
CCACCATGCCTTCAGCAAGTACCAACTTGTTGATGACATGGTTGGTTTGAC
CCTTCACTCTTGA(375bp)CTATTAGTTCCTTATTTCTCATGGAAAATCAGCCA
CCGCCGCCACCACTCCAACACCGGTTCCCTCGACCGCAACGAAGTGTTTG
TCCCAAAACCAAAATCAAAGGTATCATGGTATAACAAGTACATGAACAATC
CACCAGGGAGGGCTATCTCCCTCTTCATCACACTCACACTAGGATGGCCCT
TGTACTTGGCCTTCAATGTTTCTGGCAGACCCTATGATAGATTTGCAAGCCA
CTATGACCCTTATGCTCCCATATACTCTAACAGGGAAAGGCTTCTAATTTATG
TCTCAGATTCATCTGTCTTTGCTGTAACATATCTGCTATATCACATAGCAACT
TTGAAAGGTTTGGGTTGGGTGGTATGTGTTTATGGGGTGCCATTGCTCATTG
TGAATGGGTTTCTAGTTACCATAACCTATTTGCAGCACACACATGCATCATT
GACTCACTATGATTCATCCGAATGGGACTGGTTAAGAGGAGCATTGGCAAC
AGTGGACAGAGATTATGGGATACTGAATAAGGCATTTCATCATATAACTGAT
ACGCATGTGGCTCATCATTTGTTCTCAACAATGCCTCATTACCATGCAATGG
AAGCAACCAATGCAATAAAGCCAATATTGGGTGATTACTACCAATTTGATGG
CACCCCAGTTTACAAAGCATTGTGGAGAGAAGCCAAAGAGTGCCTCTATGT
GGAGCCAGATGATGGAGCTTCTCAGAAGGGTGTTTATTGGTACAAGAACA
AGTTCTGA
>*FAD2-A* coding sequence in normal-oleic cultivar Yueyou43
ATGGGAGCTGGAGGGCGTGTCACTAAGATTGAAGCTCAAAAGAAGCCTCT
TTCAAGGGTTCCACATTCAAACCCTCCATTCAGTGTTGGCCAACTCAAGAA
AGCAATTCCACCACATTGCTTTGAACGTTCTCTTTTCATATCATTCTCCTATG
TTGTCTATGATCTCTTAGTGGCCTACTTACTCTTCTACATTGCCACCACTTAT
TTCCACAAGCTTCCATACCCATTTTCCTTCCTTGCTTGGCCAATCTATTGGGC
CATCCAAGGCTGCATTCTCACTGGTGTTTGGGTGATTGCTCATGAGTGTGG
CCACCATGCCTTCAGCAAGTACCAACTTGTTGATGACATGGTTGGTTTGAC
CCTTCACTCTTGTCTATTAGTTCCTTATTTCTCATGGAAAATCAGCCACCGCC
GCCACCACTCCAACACCGGTTCCCTCGACCGCGACGAAGTGTTTGTCCCA
AAACCAAAATCAAAGGTATCATGGTATAACAAGTACATGAACAATCCACCA
GGGAGGGCTATCTCCCTCTTCATCACACTCACACTAGGATGGCCCTTGTACT
TGGCCTTCAATGTTTCTGGCAGACCCTATGATAGATTTGCAAGCCACTATGA
CCCTTATGCTCCCATATACTCTAACAGGGAAAGGCTTCTAATTTATGTCTCAG
ATTCATCTGTCTTTGCTGTAACATATCTGCTATATCACATAGCAACTCTGAAA
GGTTTGGGTTGGGTGGTATGTGTTTATGGGGTGCCATTGCTCATTGTGAATG
GGTTTCTAGTTACCATAACCTATTTGCAGCACACACATGCATCATTGCCTCA
CTATGATTCATCCGAATGGGACTGGTTAAGAGGAGCATTGGCAACAGTGGA

CAGAGATTATGGGATACTGAATAAGGCATTTCATCATATAACTGATACGCATG
TGGCTCATCATTTGTTCTCAACAATGCCTCATTACCATGCAATGGAAGCAAC
CAATGCAATAAAGCCAATATTGGGTGATTACTACCAATTTGATGGCACCCCA
GTTTACAAAGCATTGTGGAGAGAAGCCAAAGAGTGCCTCTATGTGGAGCC
AGATGATGGAGCTTCTCAGAAGGGTGTTTATTGGTACAAGAACAAGTTCTG
A
>*FAD2-B* coding sequence in high-oleic cultivar Yueyou271
ATGGGAGCTGGAGGGCGTGTCACTAAGATTGAAGCTCAAAAGAAGCCTCC
TTCAAGGGTTCCACATTCAAACCCTCCATTCAGTGTTGGCCAACTCAAGAA
GGCAATTCCACCACATTGCTTTGAACGTTCTCTTTTCATATCATTCTCATATG
TTGTCTATGATCTCTTAATGGCCTACTTACTCTTCTACATTGCCACCACTTATT
TCCACAAGCTTCCATACTCATTATCCTTCCTTGCTTGGCCAATCTATTGGGCC
ATCCAAGGCTGCATTCTCACCGGTGTTTGGGTGATTGCTCATGAGTGTGGC
CACCATGCCTTCAGCAAGTACCAACTTGTTGATGACATGGTTGGTTTGACC
CTTCACTCTTGTCTATTAGTTCCTTATTTCTCGTGGAAAATCAGCCACCGCC
GCCACCACTCCAACACAGGTTCCCTCA(442bp)GACCGCGACGAAGTGTTTG
TCCCGAAACCAAAATCAAAGGTATCATGGTATAACAAGTACATGAACAATC
CACCAGGGAGGGCTATTTCCCTTTTCATCACACCCACACTAGGATGGCCCT
TGTACTTGGCCTTCAATGTTTCTGGCAGACCCTATGATAGATTTGCAAGCCA
CTATGACCCTTATGCTCCCATATACTCTAACAGGGAAAGGCTTCTAATTTATG
TCTCAGATTCATCTGTCTTTGCTGTAACATATCTGCTATATCACATAGCAACT
TTGAAAGGTTTGGGTTGGGTGGTATGTGTTTATGGGGTGCCATTGCTCATTG
TGAATGGGTTTCTAGTTACCATAACCTATTTGCAGCACACACATGCAGCATT
GCCTCACTATGATTCATCCGAATGGGACTGGTTAAGAGGAGCATTGGCAAC
AGTGGACAGAGATTATGGGATACTGAATAAGGCATTTCATCATATAACTGAT
ACGCATGTGGCTCATCATTTGTTCTCAACAATGCCTCATTACCATGCAATGG
AAGCAACCAATGCAATAAAGCCAATATTGGGTGATTACTACCAATTTGATGG
CACCCCAGTTTACAAAGCATTGTGGAGAGAAGCCAAAGAGTGCCTCTATGT
GGAGCCAGATGATGGAGCTTCTCAGAAGGGTGTTTATTGGTACAAGAACA
AGTTCTGA
>*FAD2-B* coding sequence in normal-oleic cultivar Yueyou43
ATGGGAGCTGGAGGGCGTGTCACTAAGATTGAAGCTCAAAAGAAGCCTCT
TTCAAGGGTTCCACATTCAAACCCTCCATTCAGTGTTGGCCAACTCAAGAA
AGCAATTCCACCACATTGCTTTGAACGTTCTCTTTTCATATCATTCTCATATG
TTGTCTATGATCTCTTAATGGCCTACTTACTCTTCTACATTGCCACCACTTATT
TCCACAAGCTTCCATACCCATTTTCCTTCCTTGCTTGGCCAATCTATTGGGC
CATCCAAGGCTGCATTCTCACCGGTGTTTGGGTGATTGCTCATGAGTGTGG
CCACCATGCCTTCAGCAAGTACCAACTTGTTGATGACATGGTTGGTTTGAC
CCTTCACTCTTGTCTATTAGTTCCTTATTTCTCATGGAAAATCAGCCACCGCC
GCCACCACTCCAACACAGGTTCCCTCGACCGCGACGAAGTGTTTGTCCCG
AAACCAAAATCAAAGGTATCATGGTATAACAAGTACATGAACAATCCACCA
GGGAGGGCTATTTCTCTTTTCATCACACTCACACTAGGATGGCCCTTGTACC
TGGCCTTCAATGTTTCTGGCAGACCCTATGATAGATTTGCAAGCCACTATGA
CCCTTATGCTCCCATATACTCTAACAGGGAAAGGCTTCTAATTTATGTCTCAG

ATTCATCTGTCTTTGCTGTAACATATCTGCTATATCACATAGCAACTTTGAAA
GGTTTGGGTTGGGTGGTATGTGTTTATGGGGTGCCATTGCTCATTGTGAATG
GGTTTCTAGTTACCATAACCTATTTGCAGCACACACATGCATCATTGCCTCA
CTATGATTCATCCGAATGGGACTGGTTAAGAGGAGCATTGGCAACAGTGGA
CAGAGATTATGGGATACTGAATAAGGCATTTCATCATATAACTGATACGCATG
TGGCTCATCATTTGTTCTCAACGATGCCTCATTACCATGCAATGGAAGCAAC
CAATGCAATAAAGCCAATATTGGGTGATTACTACCAATTTGATGGCACCCCA
GTTTACAAAGCATTGTGGAGAGAAGCCAAAGAGTGCCTCTATGTGGAGCC
AGATGATGGAGCTTCTCAGAAGGGTGTTTATTGGTACAAGAACAAGTTCTG
A

Figure S1. Coding sequence of *FAD2-A* and *FAD2-B* in Yueyou271 (high oleic acid peanut) and Yueyou43 (normal oleic acid peanut).

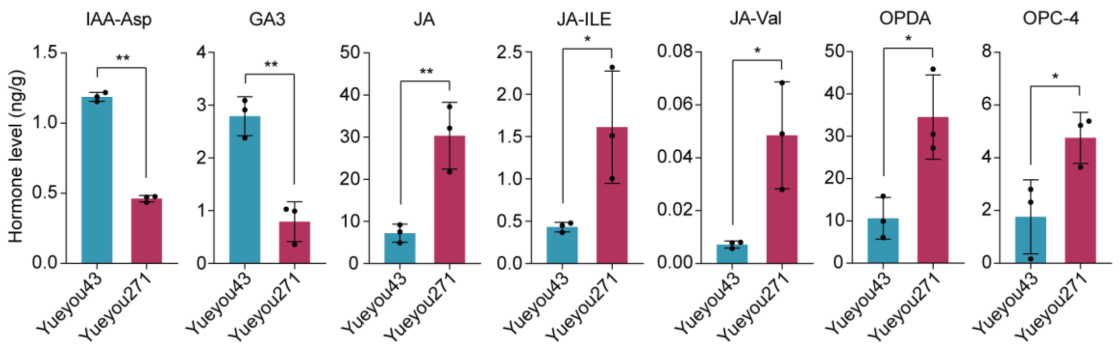

Figure S2. Phytohormone detection results in seedling leaves of Yueyou43 and Yueyou271. The histograms indicate the mean ± SD of three biological replicates. The asterisks indicate significant differences between the two varieties (T-test, *$p < 0.05$, **$p < 0.01$).

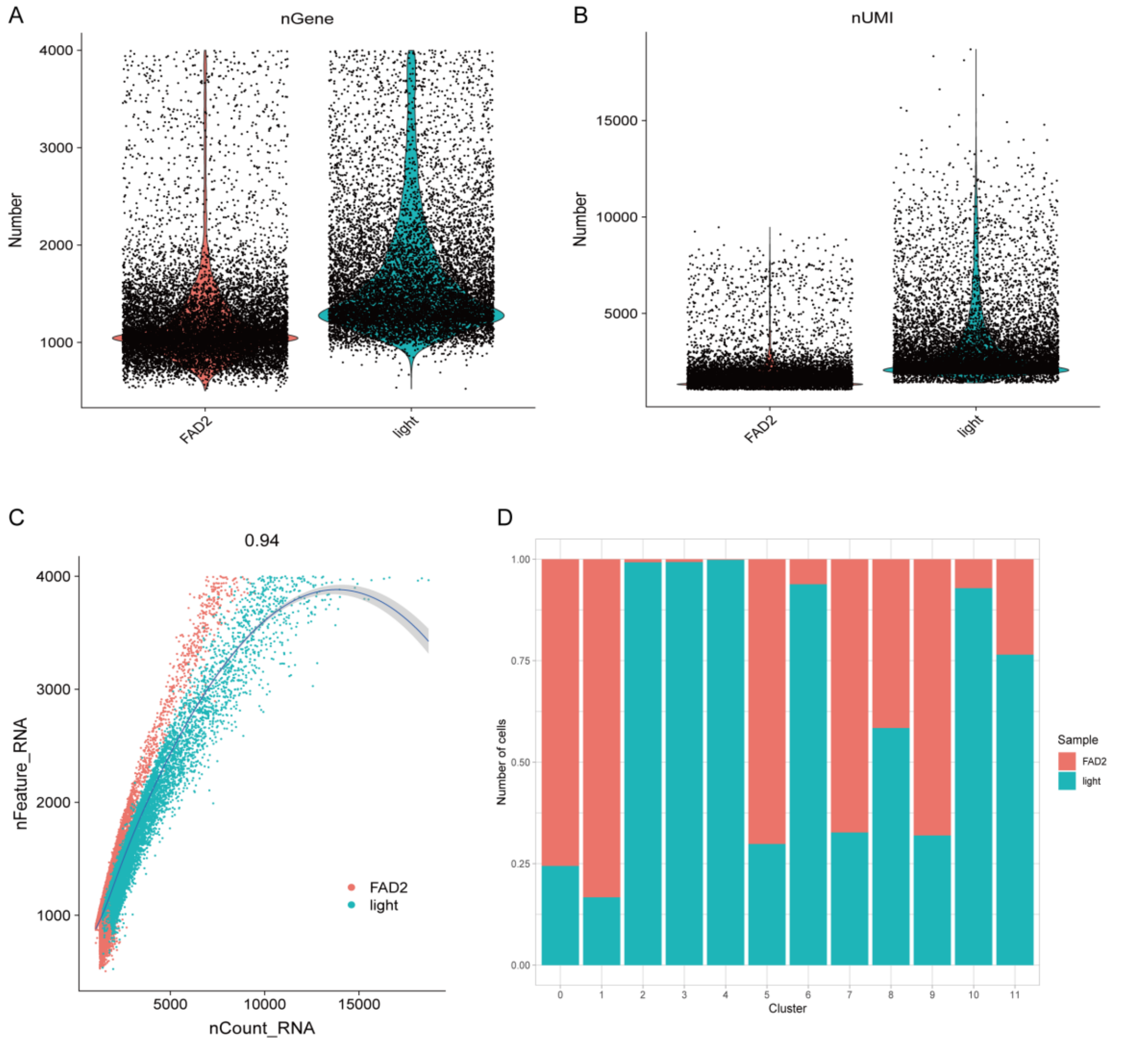

Figure S3. scRNA-seq raw data quality control. (A, B) Distribution of number of genes and UMIs. (C) Scatter plot depicting the number of genes (nFeature_RNA) and number of transcripts (nCount_RNA) per cell. (D) The cell distribution in each peanut leaf cell cluster.

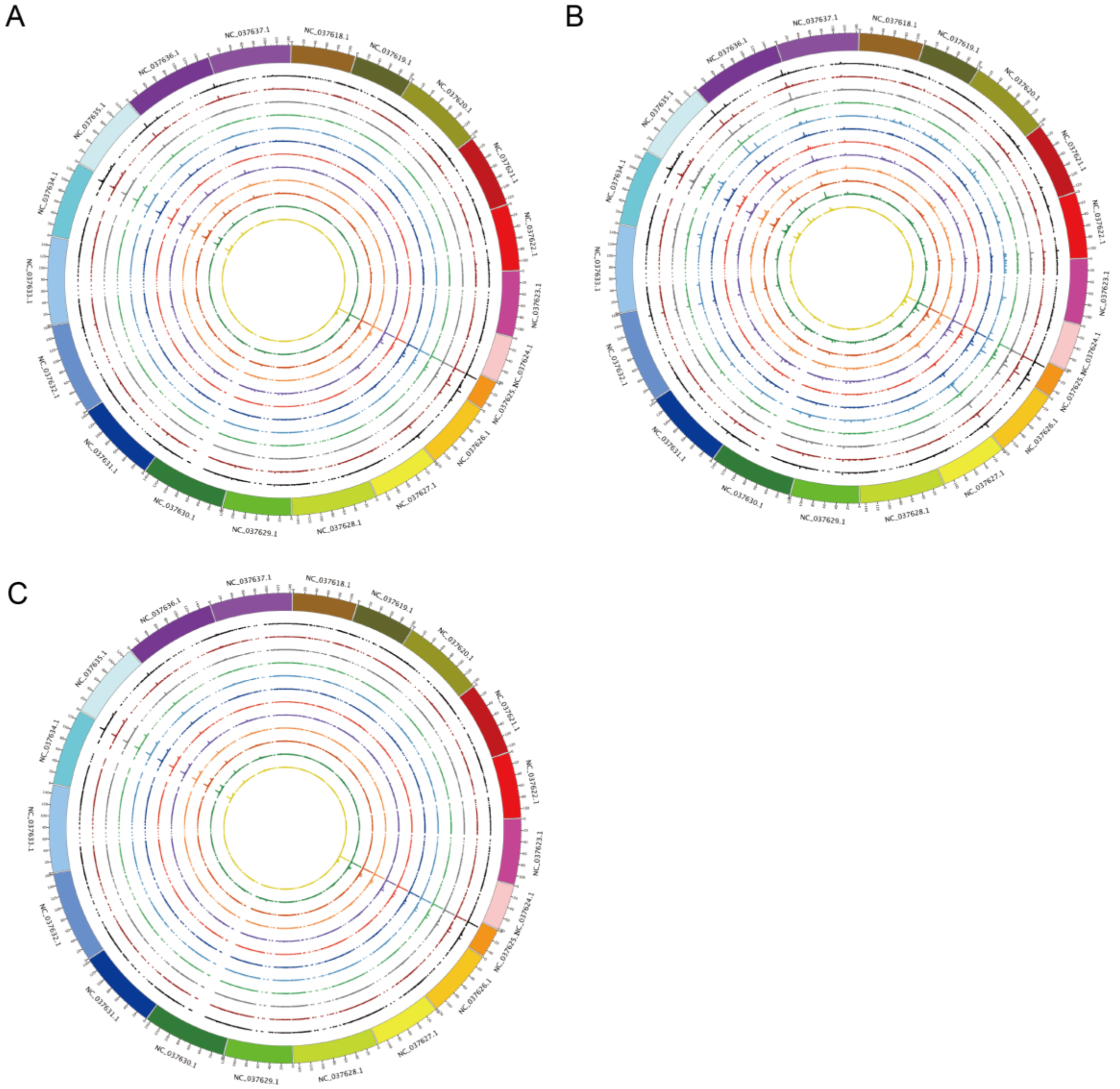

Figure S4. Circos plots depicting the single-cell gene expression pattern in leaf cells of total transcription factors (TFs). (A) Total TFs in both samples. (B) Total TFs in high-OA variety Yueyou271. (C) Total TFs in normal variety Yueyou43. From outer circular to inner circular represent the cell cluster 0 to 11.

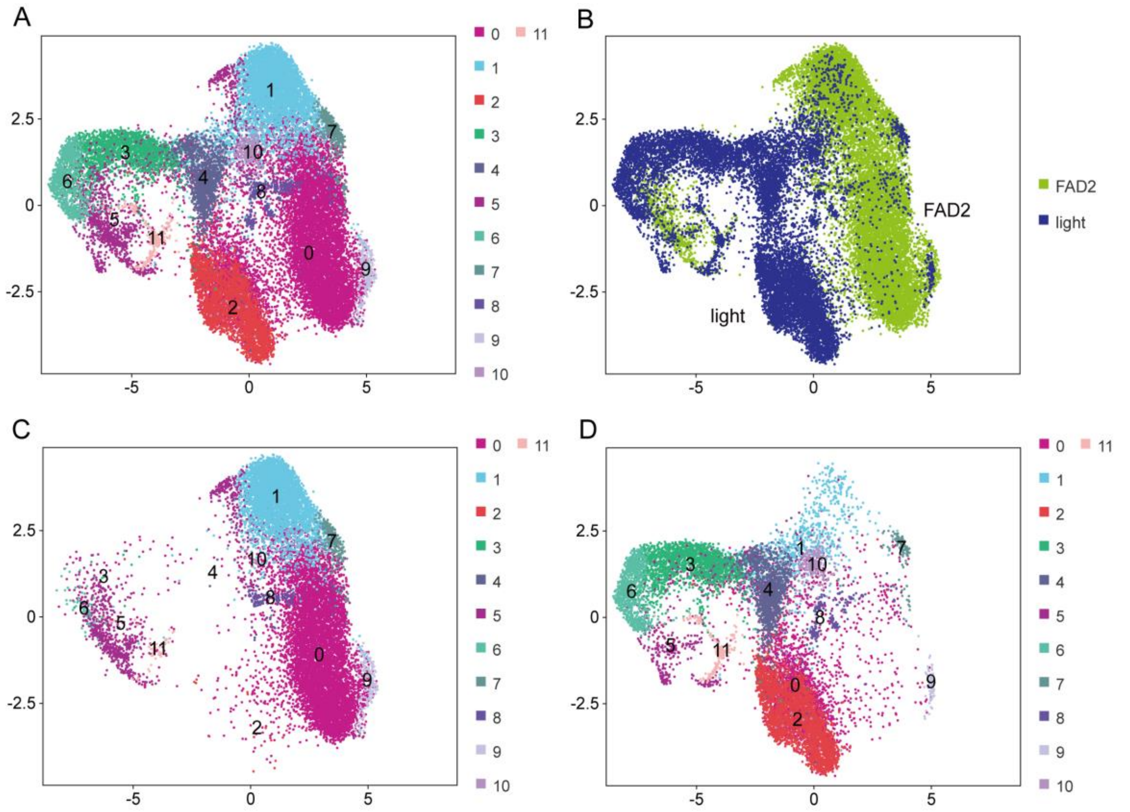

Figure S5. Visualization of 12 cell clusters using UMAP plot, each dot indicates individual cells colored based on cluster number (A) and sample ID (B). (C) and (D) are UMAP plots for cells from the *fad2* and normal peanut samples, respectively.

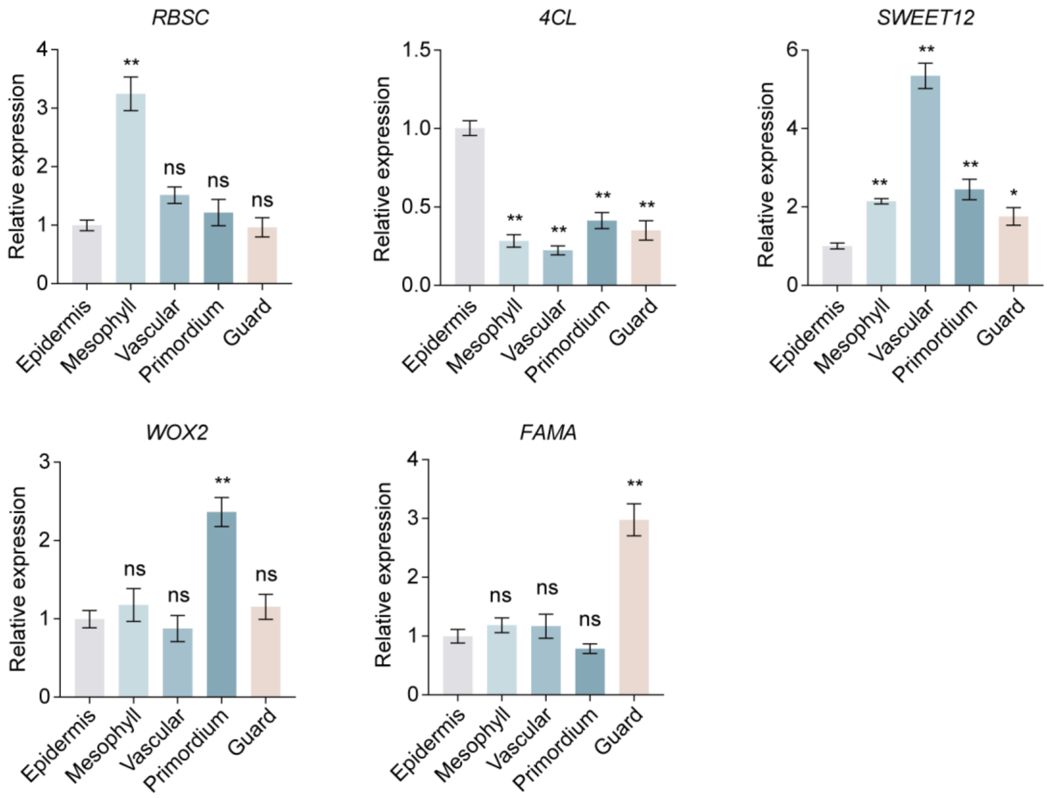

Figure S6. The validation of scRNA-seq data of the five marker genes. Histograms indicate the mean ± SD of three biological replicates. The asterisks indicate significant differences between the epidermis and other cell types. (T-test, *$p < 0.05$, **$p < 0.01$).

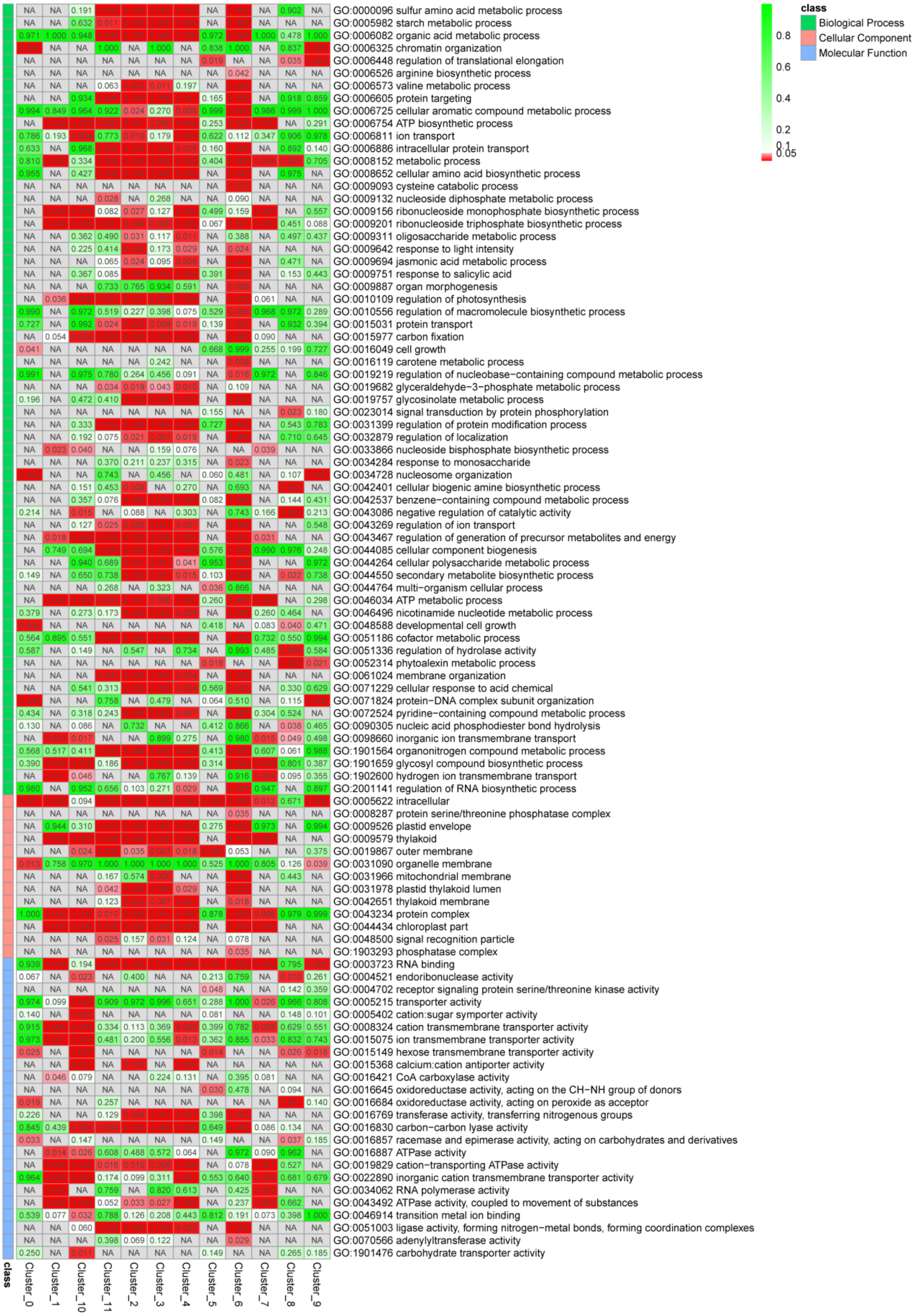

Figure S7. Go enrichment analysis of all DEGs.

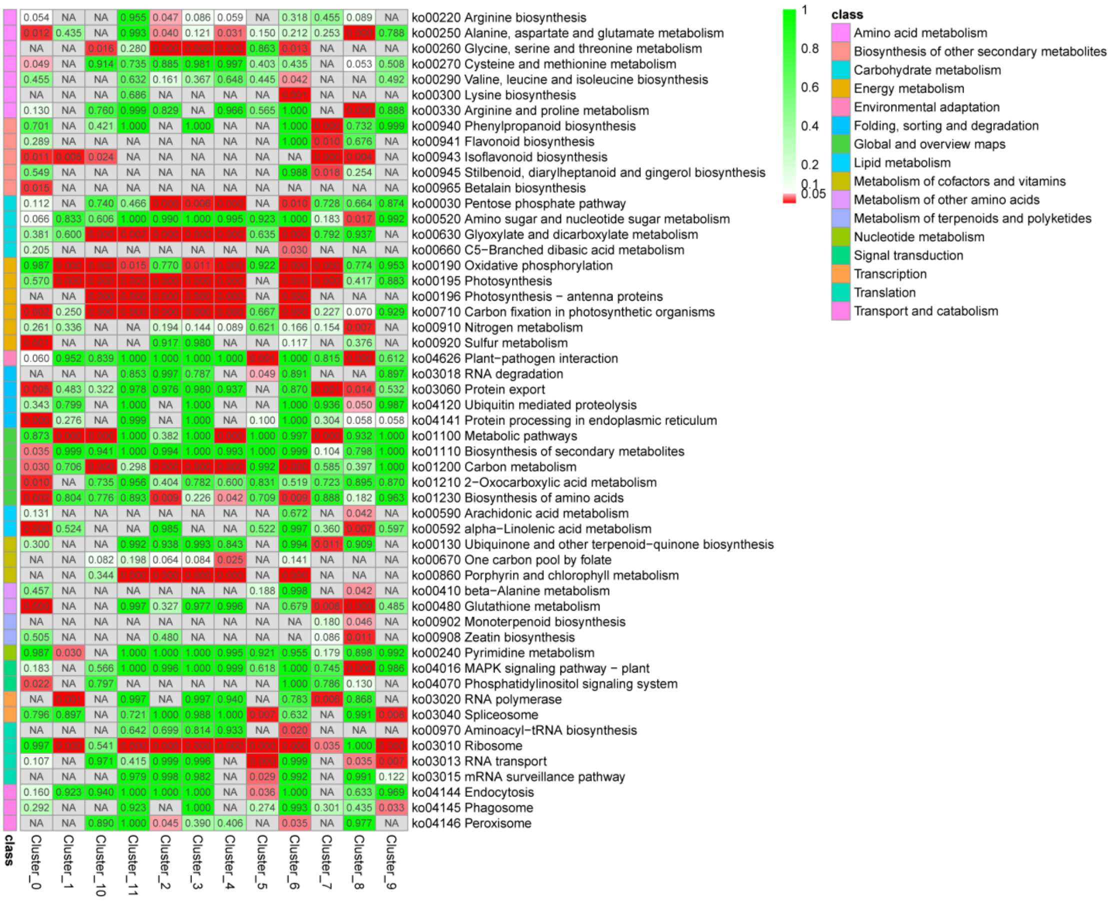

Figure S8. KEGG pathway enrichment analysis of all DEGs.

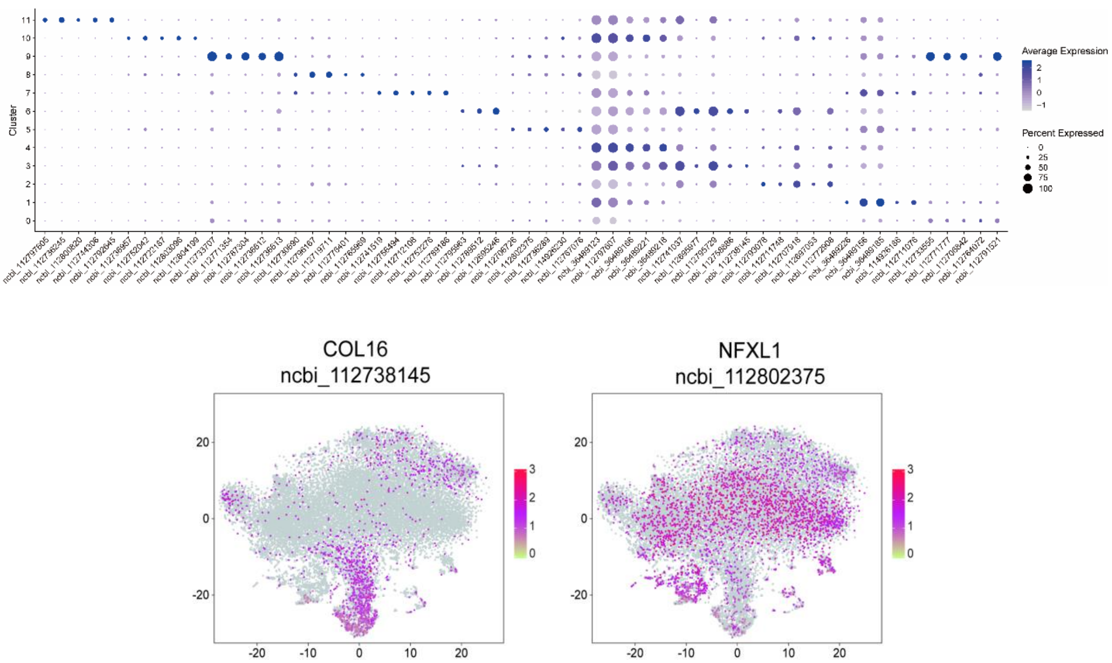

Figure S9. Dot plot depicting top five genes for each cell cluster and the cell expression distributions of two TFs (COL16 and NFXL1) in top five genes profile. Color intensity indicates the relative transcript level (average expression scale) for the indicated gene in each cell.

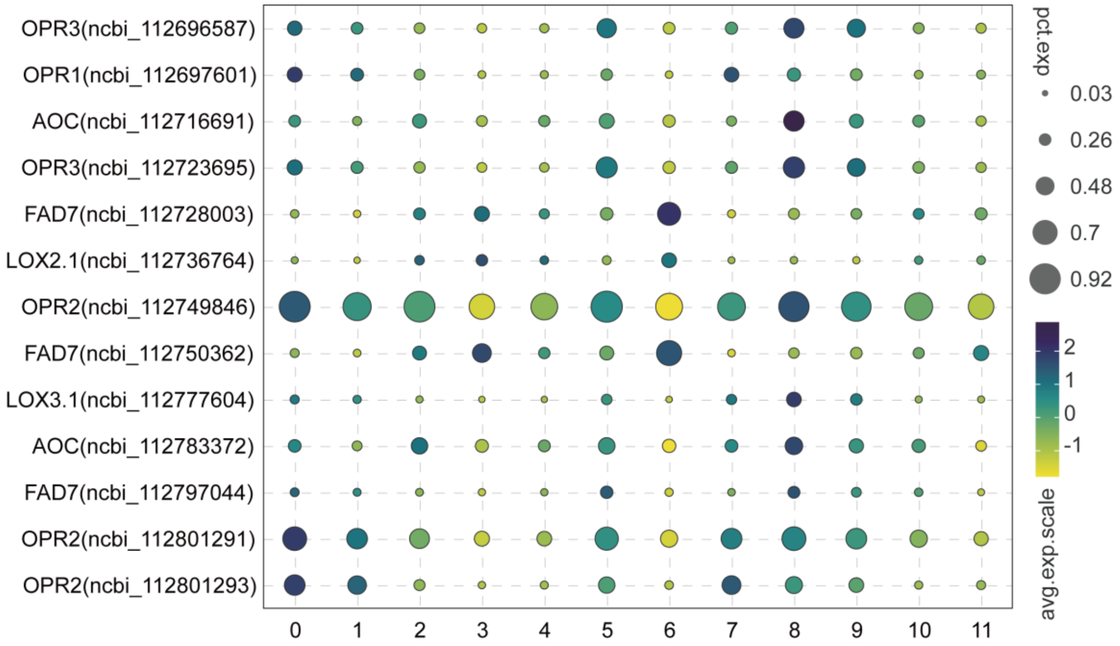

Figure S10. DEGs involved into the JA biosynthesis pathway.

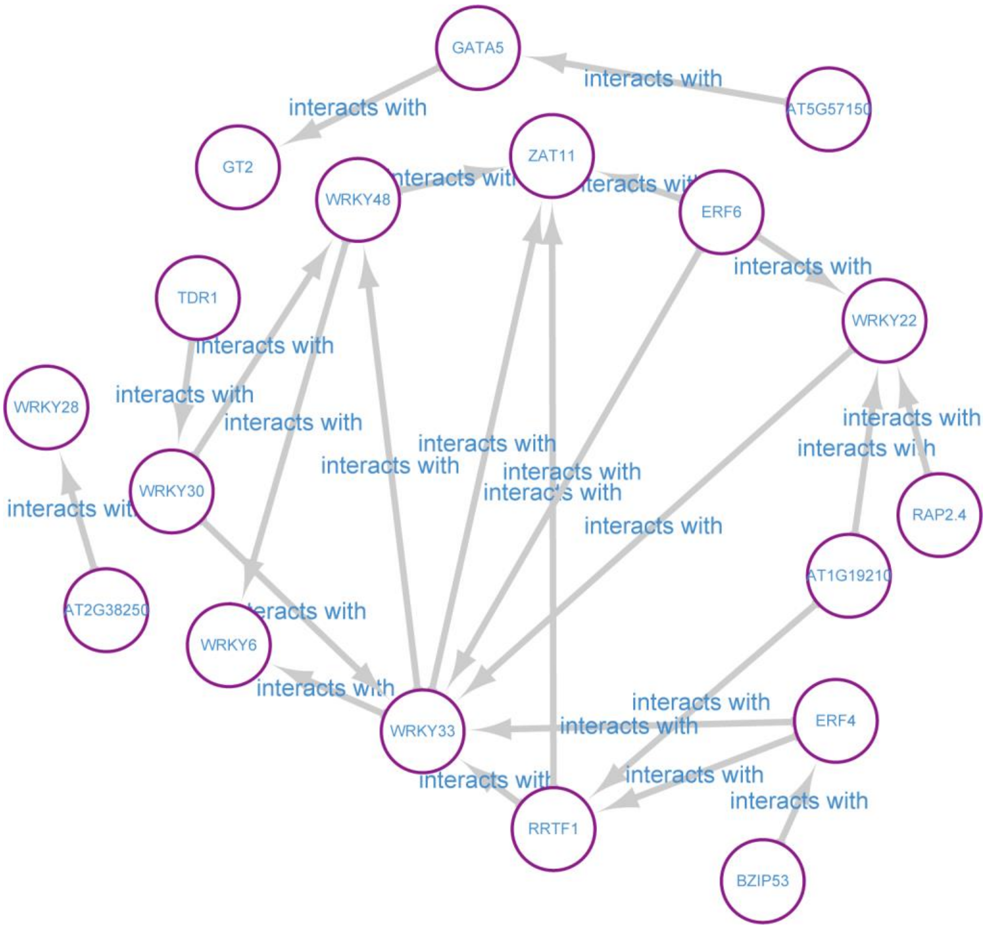

Figure S11. Interaction network of TFs constructed using *Arabidopsis* homologues of the 65 TFs in 1,649 core-DEGs profile.

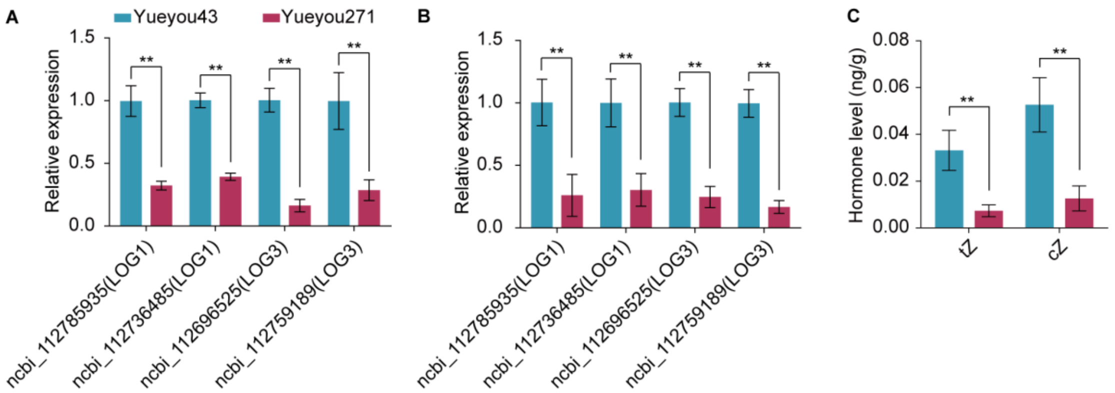

Figure S12. The validation of the scRNA-seq data in distinct cell populations. Histograms indicate the mean ± SD of three biological replicates. The asterisks indicate significant differences between the two varieties (T-test, *p < 0.05, **p < 0.01). (A) The expression level of four *LOG* genes in the leaf blade of the two varieties. (B) The expression level of four *LOG* genes in the leaf vein of the two varieties. (C) The cytokinin content in the leaf vein of the two varieties.

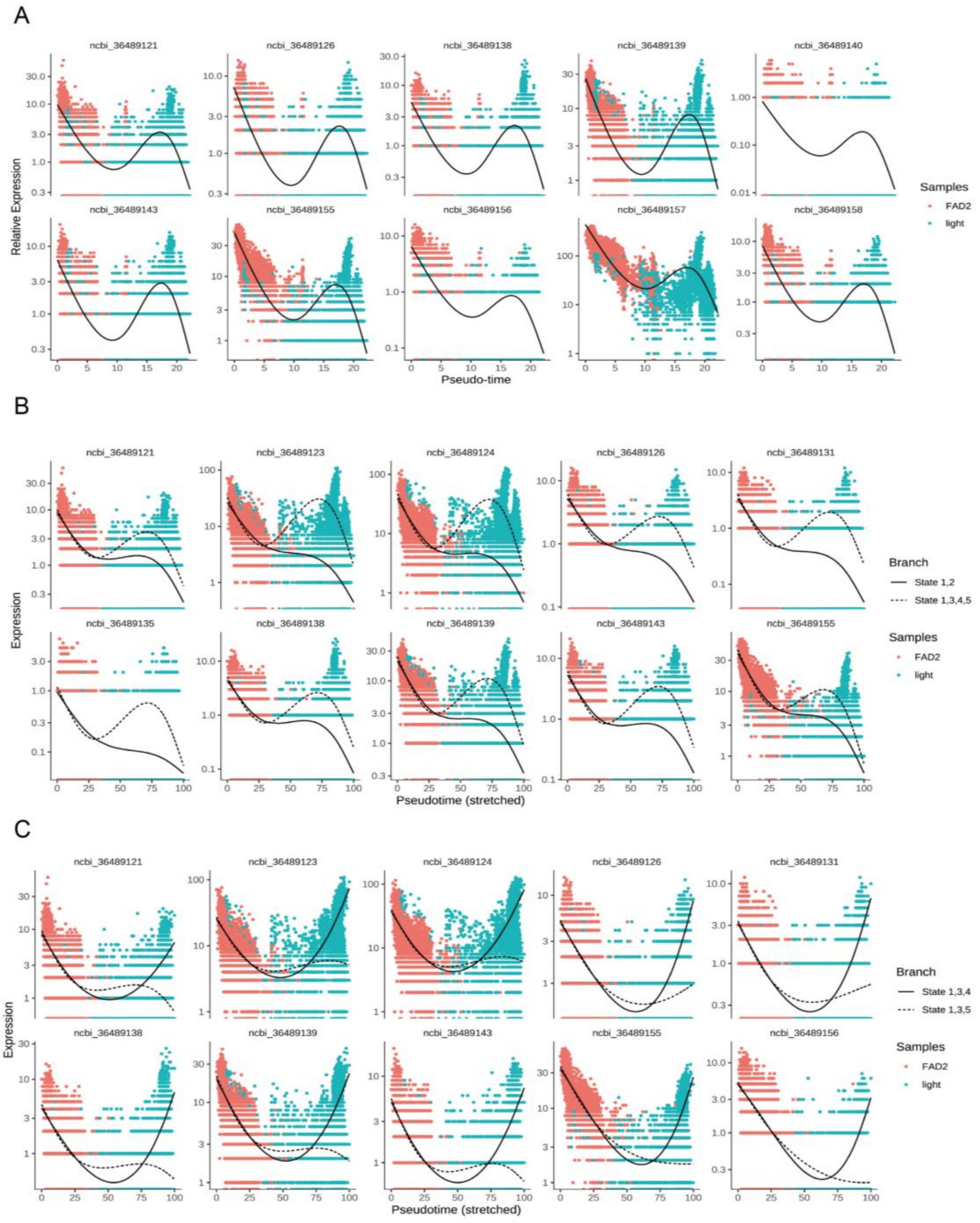

Figure S13. The relative expression of the top ten DEGs in the whole differentiation trajectory (A), cell differentiation branch 1 (B) and branch 2 (C).

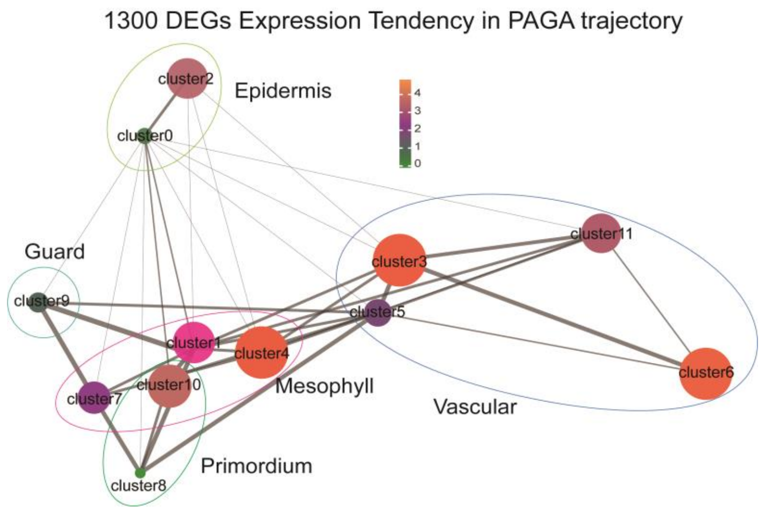

Figure S14. Expression tendency of 1,300 DEGs in PAGA trajectory.

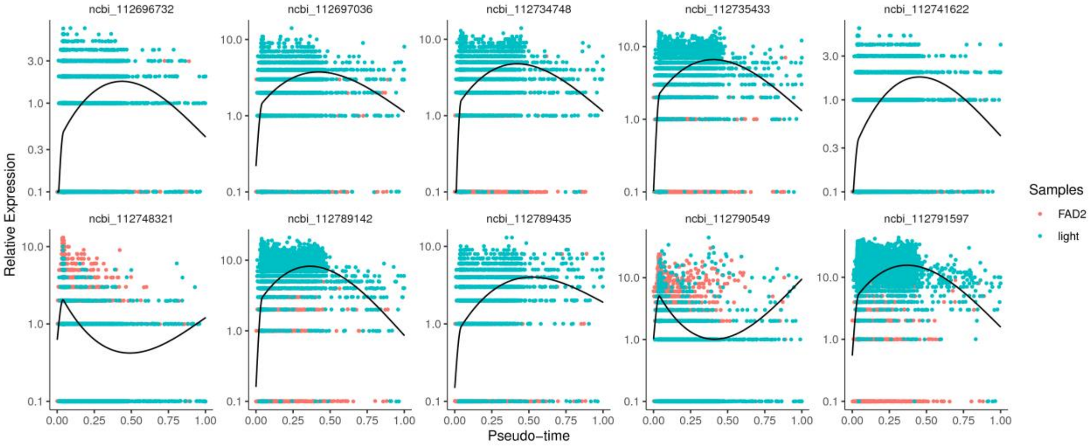

Figure S15. The relative expression of top ten DEGs in PAGA trajectory.

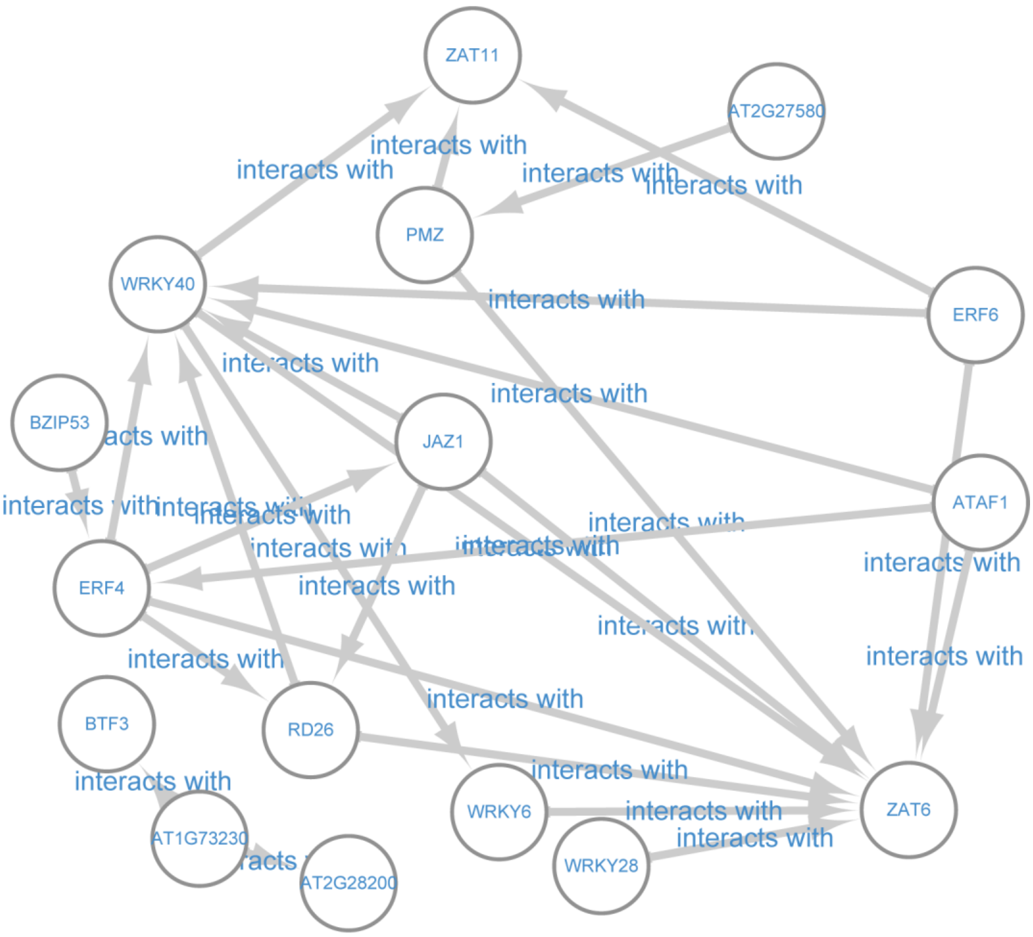

Figure S16. Interaction network of TFs constructed using *Arabidopsis* homologues of the 48 TFs in the 1,251 core-DEGs profile.

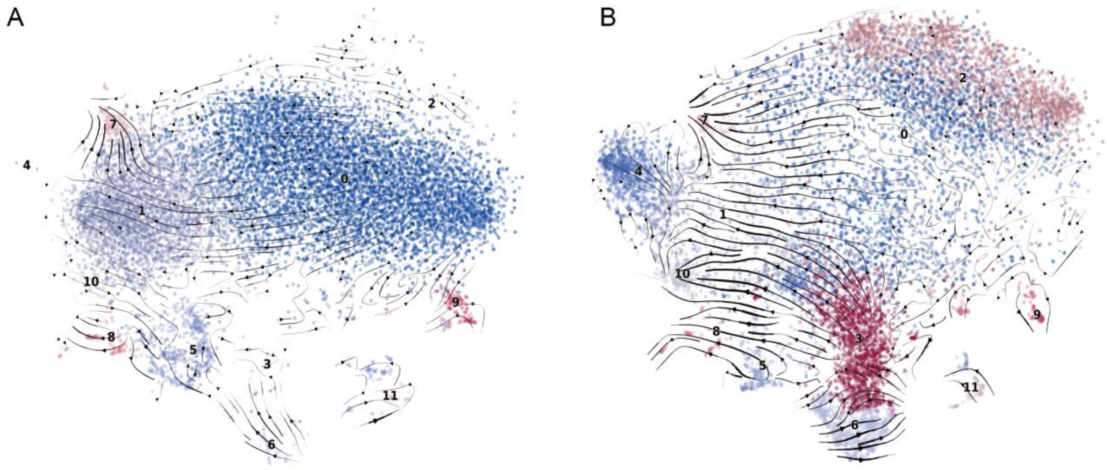

Figure S17. RNA velocity analysis of seedling leaf cells of high-OA peanut (A) and normal peanut (B).

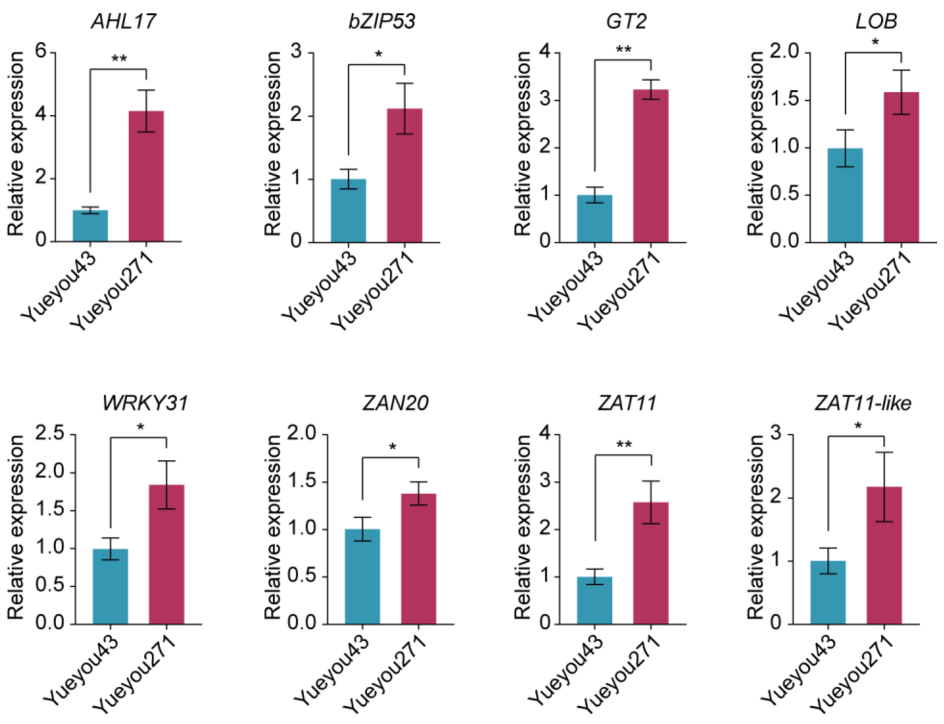

Figure S18. The validation of scRNA-seq data of the eight critical TFs identified from the 1,113 core DEGs. Histograms indicate the mean ± SD of three biological replicates. The asterisks indicate significant differences between the two varieties (T-test, $*p < 0.05$, $**p < 0.01$).
